# Corals adapted to extreme and fluctuating seawater pH increase calcification rates and have unique symbiont communities

**DOI:** 10.1002/ece3.10099

**Published:** 2023-05-29

**Authors:** Clément Tanvet, Emma F. Camp, Jill Sutton, Fanny Houlbrèque, Gérard Thouzeau, Riccardo Rodolfo‐Metalpa

**Affiliations:** ^1^ Centre IRD Nouméa UMR ENTROPIE (IRD, Université de la Réunion, Université de la Nouvelle‐Calédonie, Ifremer) Nouméa New Caledonia; ^2^ Univ Brest, CNRS, IRD, Ifremer, LEMAR Plouzané France; ^3^ Labex ICONA, International CO_2_ Natural Analogues Network Shimoda Japan; ^4^ Climate Change Cluster University of Technology Sydney Ultimo New South Wales Australia

**Keywords:** adaptation, Bouraké, calcification, coral, natural analogue, New Caledonia, ocean acidification, physiology, Symbiodiniaceae

## Abstract

Ocean acidification (OA) is a severe threat to coral reefs mainly by reducing their calcification rate. Identifying the resilience factors of corals to decreasing seawater pH is of paramount importance to predict the survivability of coral reefs in the future. This study compared corals adapted to variable pH_T_ (i.e., 7.23–8.06) from the semi‐enclosed lagoon of Bouraké, New Caledonia, to corals adapted to more stable seawater pH_T_ (i.e., 7.90–8.18). In a 100‐day aquarium experiment, we examined the physiological response and genetic diversity of Symbiodiniaceae from three coral species (*Acropora tenuis*, *Montipora digitata*, and *Porites* sp.) from both sites under three stable pH_NBS_ conditions (8.11, 7.76, 7.54) and one fluctuating pH_NBS_ regime (between 7.56 and 8.07). Bouraké corals consistently exhibited higher growth rates than corals from the stable pH environment. Interestingly, *A. tenuis* from Bouraké showed the highest growth rate under the 7.76 pH_NBS_ condition, whereas for *M. digitata,* and *Porites* sp. from Bouraké, growth was highest under the fluctuating regime and the 8.11 pH_NBS_ conditions, respectively. While OA generally decreased coral calcification by ca. 16%, Bouraké corals showed higher growth rates than corals from the stable pH environment (21% increase for *A. tenuis* to 93% for *M. digitata*, with all pH conditions pooled). This superior performance coincided with divergent symbiont communities that were more homogenous for Bouraké corals. Corals adapted to variable pH conditions appear to have a better capacity to calcify under reduced pH compared to corals native to more stable pH condition. This response was not gained by corals from the more stable environment exposed to variable pH during the 100‐day experiment, suggesting that long‐term exposure to pH fluctuations and/or differences in symbiont communities benefit calcification under OA.

## Graphical abstract

This paper investigated the physiological response and genetic diversity of Symbiodiniaceae from three coral species originating from both a stable and a fluctuating pH environment and cultured under four pH conditions for 100 days. We identified higher calcification rates and unique symbiont community for corals from the fluctuating environment. The long‐term exposure to environmental variability is thought to increase coral plasticity to the ongoing rapid ocean acidification.

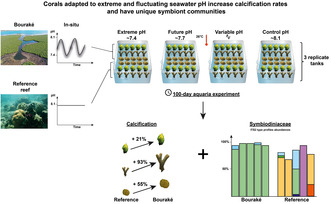

## 1 INTRODUCTION

Coral reefs have been suggested to be at risk of disappearance in the coming decades due to their sensitivity to the deadly trio of stressors including ocean warming (OW), acidification (OA), and deoxygenation (OD) (e.g., Hoegh‐Guldberg et al., 2017; Hughes et al., 2018, 2020). These stressors are expected to increase over the coming decades, leading to a dramatic decline of coral reefs by as early as 2050 (Hoegh‐Guldberg et al., 2017; IPCC Report, 2021). However, species‐specific responses to abiotic stressors (Altieri et al., 2021; Comeau et al., 2021; Jury & Toonen, 2019; Rodolfo‐Metalpa et al., 2011) and in vitro experiments (Comeau et al., 2019; Cornwall et al., 2018) have shown that some corals can be resilient to one stressor or the combination of more stressors (e.g., OA, OW, OD). Among the most likely hypotheses, it seems that chronic environmental changes could make corals more resilient to future changes in pH and/or temperature (Comeau et al., 2021; Enochs et al., 2020; Schoepf et al., 2020). Possible mechanisms that could support resilience include physiological plasticity, epigenetics, and host a more tolerant microbiome and Symbiodiniaceae (Camp et al., 2017, 2020; Putnam et al., 2016; Ros et al., 2021; Voolstra & Ziegler, 2020).

Corals thriving under variable seawater pH and temperature conditions in mangrove lagoons have shown a shift in dominant host‐associated Symbiodiniaceae taxa (Ros et al., 2021), likely contributing to the persistence of corals in such extreme environments (Camp et al., 2018, 2020; Haydon et al., 2021; Howells et al., 2012; Voolstra & Ziegler, 2020). Interestingly, some corals from stressful and/or variable environments have been associated with *Durusdinium* (formerly clade D) (Lesser et al., 2013; Schoepf et al., 2015; Stat et al., 2013; Stat & Gates, 2011; Wall et al., 2021) that is considered a ‘stress‐tolerant’ taxa, although this is not always the case (Camp et al., 2019, 2020). Whilst corals with a stable host‐Symbiodiniaceae association have been suggested to be physiologically more resilient under stressful conditions (Grottoli et al., 2018; Howells et al., 2020), it remains relevant to explore how stability (i.e., fidelity) and flexibility in the Symbiodiniaceae diversity benefit host fitness under stress conditions (Fabina et al., 2013; McIlroy et al., 2020; Putnam et al., 2012; Smith et al., 2017; Ziegler et al., 2015).

More generally, coral resilience to extreme conditions includes trade‐offs in the main physiological traits, such as reduced calcification rate (Bay & Palumbi, 2016; Camp et al., 2020; Cunning et al., 2015) and photosynthesis (Camp et al., 2020), increased photodamage (Silverstein et al., 2017), and change in the autotrophic nutrition capacities (Wall et al., 2020). For instance, corals with thermotolerant symbionts have been reported to resist bleaching but grow more slowly (Jones & Berkelmans, 2010). Most of these trade‐offs remain poorly tested on corals already adapted to conditions close to or even worse than the ones predicted for the end of this century. Yet, extreme environments, such as mangrove lagoons have been identified to be faithful natural laboratories for studying species living under the combination of future extreme conditions in OA, OD, and OW (Camp et al., 2018).

The semi‐enclosed lagoon of Bouraké in New Caledonia is one of these extreme environments which has been recently environmentally and ecologically characterized (Camp et al., 2017; Maggioni et al., 2021; Tanvet et al., 2022). Bouraké has fluctuating acidified, warm, and deoxygenated conditions, although a highly‐diversified coral reef develops (Camp et al., 2017). The environmental variability in the dissolved oxygen, temperature, and pH in Bouraké is directly related to the tidal cycle with changes on a single day by up to 4.91 mg O_2_ L^−1^, 6.50°C, and 0.69 pH_T_ units (Maggioni et al., 2021), respectively. Such environmental conditions likely enhanced coral respiration rates and total chlorophyll content, while reducing photosynthesis and calcification rates (Camp et al., 2017; Jacquemont et al., 2022). In addition to the particular physiological response of some corals from Bouraké, Camp et al. (2020) also found differences in host‐microorganisms associations (Symbiodiniaceae and bacteria) between Bouraké and an adjacent reef. Although species‐specific, the differences in the Symbiodiniaceae found in three coral species (Camp et al., 2020) highlight the potential role that Symbiodiniaceae play in influencing coral holobiont resilience to environmental stress (Sampayo et al., 2008). Corals in Bouraké have likely either adapted to, or sustain key trade‐offs to achieve resilience in an environment somewhat reproducing future climate conditions.

In the present study, we conducted a 100‐day OA experiment in aquaria using three coral species (*Acropora tenuis*, *Montipora digitata*, and *Porites* sp.) from the highly variable Bouraké and a less variable adjacent reference reef to assess: (i) whether corals adapted to ambient or fluctuating pH conditions alter their metabolic rates and calcification under different levels of OA (i.e., 7.54, 7.76, 8.11, and 7.56–8.07), and (ii) whether Symbiodiniaceae communities are distinct between habitats and treatments at the end of the experiment. In addition, we considered both static pH_NBS_ (7.54 ± 0.08; 7.76 ± 0.07; 8.11 ± 0.05) and variable (ranging from 7.56 ± 0.07 to 8.07 ± 0.07) pH_NBS_ conditions to assess the role that fluctuating pH may play in the success of Bouraké corals, and whether corals adapted to stable pH can acclimate when exposed during 100 days to variable pH. We hypothesize that corals from Bouraké exhibit enhanced physiological traits and a distinct Symbiodiniaceae community, compared with corals from the less variable reef, and that the natural diel fluctuations in seawater pH promote their resilience to OA.

## 2 MATERIALS AND METHODS

### 2.1 Study sites, coral collection, and acclimation

All corals were collected at the end of January 2020 in the semi‐enclosed lagoon of Bouraké, New Caledonia (21°57′3.971″ S 165°59′24.233″ E) (Figure S1), and at one reference reef adjacent to Bouraké (21°58′6.966″ S 165°56′43.907″ E) (St R1, 4 km away). Environmental conditions at both sites have been previously characterized across several days in February, March, and May 2016 (Camp et al., 2017) and across numerous surveys (from 22 to 72 semidiurnal tidal cycles, depending on environmental parameters and site) from 2017 to 2020 (Maggioni et al., 2021). In Bouraké, dissolved oxygen (DO) and pH regularly fluctuated according to the tide (ca. 1 m tidal range), from 1.87 to 7.24 mg O_2_ L^−1^ (mean of 5.23 ± 0.89 mg O_2_ L^−1^ recorded during 42 semidiurnal tidal cycles from 2016 to 2020) and from 7.23 to 8.06 pH_T_ (mean of 7.67 ± 0.23 pH_T_ recorded during 72 semidiurnal tidal cycles from 2016 to 2020), respectively (Maggioni et al., 2021). Seawater temperature varied from 17.50°C in winter to 33.80°C during summer, when the temperature might change by up to 6.50°C in a single day (mean of 25.63°C ± 2.85°C during a continuous record from January 2019 to April 2020 with 10‐minute interval) (Maggioni et al., 2021). In comparison to Bouraké, DO, pH, and temperature were more stable at the reference site (hereafter reference) during equivalent periods with means of 6.45 ± 0.95 mg O_2_ L^−1^, 8.02 ± 0.04 pH_T_, and 25.25°C ± 1.89°C, respectively (Maggioni et al., 2021). For example, at the end of the austral summer from February to March 2016, the reefs in the Bouraké lagoon spent 44% of the time at pH_T_ of 7.7–7.8, and 71% of the time at temperatures predicted for the end of the century under scenario RCP7.0 (Camp et al., 2017). Notwithstanding such extreme and chronic conditions, 66 coral species form reefs all along the Bouraké lagoon (Maggioni et al., 2021).

Fifteen mature colonies (30–60 cm diameter) of the branching *A. tenuis* (Dana, 1846) and *M. digitata* (Dana, 1846), and the massive *Porites* sp. were sampled at each site at ca. 2‐meter depth. Only colonies at a distance of at least 5 m from each other were collected to limit the risks of clonality in our sampling. From each branching colony, four terminal portions of branches (ca. 3–5 cm long) were collected using pliers. For *Porites* sp., four samples (mean of ca. 8 cm^2^) were collected from each colony using a 3‐cm‐diameter steel tube and a hammer. Coral fragments were transported in a cooler to the “Aquarium des Lagons” (Nouméa), which is 2 h distant from Bouraké, in individual hermetic zip bags (one for each colony of each species) containing seawater from the collection site. At the laboratory, fragments of *A. tenuis* and *M. digitata* were attached to nylon wires and suspended in two 200 L tanks, one for each site of collection (see below). Fragments of *Porites* sp. were mounted on labeled 2 × 2 cm PVC plates using epoxy resin (Holdfast, Aquarium Systems) and placed at the bottom of the tanks. The exposed skeleton was covered with the resin to avoid turf algae proliferation and potential skeletal dissolution. All 360 fragments (2 sites, 3 species, 15 colonies per species, 4 samples per colony, resulting in a base replication of *n* = 15 per treatment) were allowed to recover for 3 weeks in the two flow‐through aquaria (see below for more details) settled at the same temperature (26.0°C ± 0.5°C) recorded in situ at the time of collection. Seawater pH and carbonate chemistry replicated mean values of Bouraké and reference sites (ca. 7.7 and 8.1 pH_NBS_ units, respectively). Temperature and pH were kept constant using heaters and bubbling pure CO_2_ in each tank. All tanks were connected to an IKS logger system (IKS, AquaStar, accuracy ±0.05 pH unit and 0.1°C) continuously monitoring but not recording the pH conditions. During the recovery period, coral fragments received the same lighting intensity (ca. 250 μmol photons m^2^ s^−1^) using four Aquablue Plus neon bulbs (15.000 K, Giesemann), and they were fed once a week with freshly hatched *Artemia salina* nauplii (regular equivalent concentrations tossed in the tanks; Houlbrèque et al., 2015). At the end of the 3 weeks recovery period, the naked skeleton of branching corals was already covered by the tissue, while new tissue was visible on the resin embedding *Porites* sp.

### 2.2 Aquarium setup and physiological measurements

#### 2.2.1 Aquarium conditions and experimental set‐up

At the end of the recovery period, corals from each site of collection (called origin hereafter) were assigned to one of the 12 experimental tanks as follows. Three replicate 10 L tanks were set up for each of the four conditions of pH (monitored with an IKS logger system and pH Metrohm in NBS scale) (Table 1) with averages recorded during the 100‐day experiment: (i) stable pH (Control; pH_NBS_ 8.11 ± 0.05; *p*CO_2_ 474 μatm); (ii) future reef pH based on the RCP7.0 IPCC scenario (IPCC Report, 2021) (Future; pH_NBS_ 7.76 ± 0.07; *p*CO_2_ 1192 μatm); (iii) extreme stable pH (Extreme; pH_NBS_ 7.54 ± 0.08; *p*CO_2_ 2115 μatm); and (iv) variable pH reproducing the semidiurnal tide phase variation of pH in Bouraké with four peaks of pH on a 24‐h basis, two at low and two at high pH (Variable; mean pH_NBS_ ranging from 7.56 ± 0.07 to 8.07 ± 0.07; *p*CO_2_ ranging from 1968 to 533 μatm, respectively). While extreme stable pH was arbitrarily chosen, control and variable pH were settled based on the mean and variance data collected respectively at reference and Bouraké reef between 2016 and 2020 (see Maggioni et al., 2021). Five fragments per coral species (*n* = 3) and per origin (*n* = 2) were randomly positioned in each tank. *Porites* samples were positioned on the bottom of the tanks while the branching corals were suspended.

**TABLE 1:** Seawater parameters measured during the 100‐day experiment, and carbonate chemistry calculated for each pH condition (the three replicated tanks were pooled)

Condition	Measured			Calculated			
	Temp	pH	*p*CO_2_	DIC	HCO_3_	CO_3_^2−^	Ω_arag_
	(°C)	(NBS)	(μatm)	(μmol kg^−1^)	(μmol kg^−1^)	(μmol kg^−1^)	
Control							
Mean	26.23	8.11	479	1926	1731	182	2.89
SD	0.49	0.05	72	30	47	19	0.30
Min	24.20	7.96	300	1834	1587	136	2.16
Max	27.50	8.27	717	2002	1847	239	3.79
Future							
Mean	26.18	7.76	1207	2097	1970	94	1.49
SD	0.52	0.07	198	26	34	14	0.22
Min	24.20	7.63	622	1993	1823	69	1.10
Max	27.50	8.01	1679	2148	2032	152	2.43
Extreme							
Mean	26.33	7.54	2141	2173	2056	58	0.93
SD	0.50	0.08	381	26	27	11	0.17
Min	23.90	7.31	683	2009	1849	35	0.55
Max	28.30	7.98	3610	2249	2115	141	2.24
Variable							
Mean	26.39	7.75	1427	2090	1951	101	1.60
SD	0.58	0.23	710	93	124	50	0.79
Min	24.20	7.37	370	1893	1671	40	0.63
Max	28.30	8.20	3181	2230	2103	212	3.36

*Note*: Seawater carbonate chemistry was calculated using mean values for *A*_T_ (2187, 2204, 2202, and 2202 μmol kg^−1^ for Control, pH_NBS_ 8.11; Future, pH_NBS_ 7.76; Extreme, pH_NBS_ 7.54; and Variable, pH_NBS_ 7.56–8.07, respectively), and mean salinity value of 35.61.

Seawater in the tanks was renewed at a rate of 16.5 L h^−1^ (renewal rate of ca. 165% h^−1^) and mixed using a submersible pump (micro‐jet MC 320, Aquarium system). Each tank was supplied with seawater pumped from one to three 60 L sump tanks settled at different pH (7.4, 7.7, and 8.1). In addition, three more sump tanks were set up respectively at pH 7.6, 7.8, and 7.9 and were used to better simulate the pH variation in the variable treatment (see below). Each sump tank was continuously supplied with 50 μm‐filtered seawater pumped at 5 m depth in front of the Aquarium des Lagons (Baie des Citrons, Nouméa). Each sump contained a submersible pump (23 W Eheim), a heater, and an external refrigerating system, both connected to an IKS logger system (IKS, AquaStar), and temperature and pH probes. Seawater pH_NBS_ and temperature values were continuously monitored but not recorded by an IKS pH logger and daily verified (randomly during the day) using a portable pH‐meter (Metrohm 826 coupled with a LL Aquatrode Plus SC, sensitivity of 0.001 pH units), both calibrated using NBS solutions (pH 4.0 and 7.0 from the Seawater National Bureau of Standards). Sumps were all maintained at 26.3°C ± 0.7°C, and at one of the 6 pH conditions, automated by IKS (AquaStar). Seawater pH was set to the desired value by bubbling pure CO_2_ gas. Each experimental tank received seawater at the assigned temperature and pH from the respective sump tank. In contrast, for the variable pH treatment, the time function of the IKS system was used to simulate the pH changes measured at Bouraké, according to a semidiurnal tide cycle with high pH in the middle of the day/night (01:00–04:00 am/pm) and a low pH at the beginning of the day/night (08:00–11:00 am/pm). Briefly, tanks received seawater either from one or simultaneously from different sumps according to a predetermined timetable (Table S1; Figure S2). Irradiance in all tanks increased from 0 to ca. 250–300 μmol photons m^−2^ s^−1^ on a 12:12 h light:dark cycle (06:00–18:00 h lighting vs. 18:00–06:00 h darkness; ramp until 10:00 h and decline from 14:00 h) using LED lights (Mitras LX6100, GHL; see Table S2 for led brightness wave simulation) according to Biscéré et al. (2019), Houlbrèque et al. (2015) and Jacquemont et al. (2022). Irradiance was monitored during 48‐h cycles using an NKE PAR with LI‐193 spherical quantum sensor and showed maximum values ranging from ca. 270 to 320 μmol photons m^−2^ s^−1^ (Figure S3). Tanks' positions were repeatedly changed during the duration of the experiment to minimize any potential spatial variability in the light intensity received by corals. A Seabird SeaFET pH logger and YSI 600 OMS‐M probes were used to measure over 48‐h cycles seawater temperature, pH_T_, salinity, and dissolved oxygen (DO) in the experimental tanks with the intention to document the variation in such a parameter over repeated diel cycles. Colonies were fed with freshly hatched *Artemia salina nauplii* once a week (regular equivalent concentrations tossed in the tanks) and were maintained under these experimental conditions for 100 days. Tanks, resins, and wires were regularly cleaned for turf algae proliferation.

Total alkalinity (*A*_T_) was measured twice a month at various daytime to have the average total alkalinity reflecting the different pH values on each of the 12 tanks over the experiment. For that, water samples were filtered at 0.45 μm (GF/F Whitman), preserved with saturated HgCl_2,_ and stored in the dark at 4°C to avoid biological alteration. Two 20‐mL replicates were analyzed using an auto titrator (Eco Titrator, Metrohm), and *A*_T_ was calculated from the Gran function. Results were corrected against *A*_T_ standards (Andrew G. Dickson, batch no. 155, Scripps, USA). Dissolved inorganic carbon (DIC), *p*CO_2,_ and saturation states of aragonite (Ω_arag_) were calculated with the software CO2SYS, using pH (NBS scale), *A*_T_, temperature, values for the K1 and K2 carbonic acid constants from Lueker et al. (2000), for KHSO_4_ from Dickson (1990), for KHF from (Perez & Fraga, 1987), for [B]_I_ from (Lee et al., 2010), and an averaged atmospheric pressure of 1.015 atm (source: Meteo France).

Nutrient content was measured twice a month on each of the 12 tanks. The sampling of nutrients (orthosilicic acid [Si(OH)_4_], nitrogen oxide [NO_x_] (i.e., the sum of NO_2_^−^ and NO_3_^−^), and phosphate [PO_4_]^3−^) was done using 20 mL polypropylene vials, rinsed three times using filtered seawater (Whatman™ Puradisc CA syringe filters 0.45 μm), filled with the sample, and immediately poisoned with 20 μL saturated HgCl_2_. Measurements of PO_4_^3−^, NO_x,_ and Si(OH)_4_ were performed by colorimetry (Seal Analytical).

#### 2.2.2 Growth rate

After the recovery period and at the end of the experiment, each coral fragment was weighed using the buoyant weight technique (Davies, 1989). Samples were weighed using a Sartorius ENTRIS 224i‐1S electronic balance (readability 0.1 mg) in seawater of known density (calculated from temperature and salinity, both measured in each 10 samples). Dry skeleton weight was calculated using the density of pure aragonite (2.94 g cm^−3^); growth rates (in terms of calcification rates) were calculated as the change in dry weight between the initial (T_0_) and the final weight (T_F_) during the experiment and expressed either in mg g^−1^ d^−1^ (normalized per weight of CaCO_3_) or mg cm^−2^ d^−1^ (normalized per surface area of the coral, see below) depending on the species.

#### 2.2.3 Photosynthetic efficiency

During the last week of the 100‐day experiment, maximum photochemical efficiency (*F*_v_ */F*_m_) and the relative electron transport rate (rETR) of the photosystem II (PSII) of symbionts *in hospite* were measured on all corals using a pulse amplitude modulation (PAM) fluorometer (DIVING‐PAM, Waltz; Schreiber et al., 1986). Before measurements, coral fragments were dark adapted for 15 min (Hoegh‐Guldberg & Jones, 1999). Measurements were performed in dark (no light source apart from the use of a red headlamp); the 8 mm optical fiber was maintained perpendicular to the fragment's surface using a black jacket at a fixed distance of 5 mm. Rapid‐light curves used a measure of ETR, determined as follow (rETR = Δ*F/F'*_*m*_ × 0.5 × PAR) with nine steps of fluorescence measurements (4, 120, 191, 278, 376, 546, 729, 1078, 1544 μmol photon m^−2^ s^−1^) according to Ralph et al. (1999). Because corals were dark adapted we considered the first point the maximal quantum yield *F*_v_ */F*_m_. RLC uses a very short illumination period, which was found to be not correct to measure the real ETR (see Enríquez & Borowitzka, 2010). Because we could not perform long RLC on the 135 corals, we limited our analysis to the maximum rETR_max_, assuming this limitation for all corals and conditions. Comparisons between treatments and origin were done on the maximum quantum yield *F*_v_ */F*_m_ and the maximum rETR_max_.

#### 2.2.4 Photosynthesis and respiration rates

At the end of the experiment, seven corals for each pH condition (*n* = 4), each origin (*n* = 2), and each species (*n* = 3) were randomly selected (*n* = 168 total) and their oxygen production and consumption rates were measured daily in the light and dark during four consecutive days. Corals were individually placed in 100 mL Pyrex glass beakers which were filled with seawater from their respective tank treatment and hermetically sealed underwater with transparent cellophane and a rubber band (Jacquemont et al., 2022). Two control beakers without coral fragments were used to measure any metabolic activity of microbes in the water. We were able to process 28 samples simultaneously (plus two empty beakers) using two 15‐place magnetic stirring plates (Telesystem 15, Thermo Scientific). The 30 beakers were semi‐immersed in a water bath positioned above the stirring plates and settled at a temperature of 26.0°C ± 0.5°C using one heater and two submersible water pumps to homogenize water temperature. Each beaker had one O_2_ sensor spot (PreSens) fixed on the glass and contained a stirring bar and a bridge made of plastic mesh to separate the coral fragment from the bar. Corals were first incubated in the light for 50 min under ca. 250 μmol photons m^2^ s^−1^ and then in the dark for the same time (Biscéré et al., 2019; Jacquemont et al., 2022). Incubation time was preliminary defined to avoid both hyperoxic and hypoxic conditions in the beakers. Temperature and dissolved oxygen (DO) in mg L^−1^ were measured at the beginning and the end of each incubation in each beaker using an optical fiber (PreSens Fibox 4 trace). Before measurement, corals were left for 10 min under either light or dark conditions and then DO was measured. At the end of the light incubation, each beaker was opened and seawater volume was measured using a graduated cylinder to normalize the DO concentration to the seawater volume of each incubation. Fresh seawater from the corresponding experimental tank was then added before the beakers were resealed and placed on the stirring plates under dark conditions. At the end of each incubation pair (dark and light) for all pH conditions, coral fragments were frozen at −20°C for further analysis of the chlorophyll, symbiont density, symbiont community analysis, and protein contents, and to determine their skeletal surface area.

Rates of net photosynthesis (*P*_net_) and respiration in the dark (*R*_dark_) were calculated using the change in DO concentrations in each beaker corrected by the mean of the microbial activity measured in the two empty beakers, and normalized by the incubation duration (hours), the volume of seawater in each beaker (L), and the coral's surface (cm^2^). Rates of gross photosynthesis (*P*_g_) were calculated as:

(1) $$P_{g}=P_{\mathrm{net}}+|R_{\text{dark}}|$$

Data were normalized per surface area of the fragment as described below. *P*_net_, *P*_g,_ and *R*_dark_ are expressed in mg O_2_ cm^−2^ h^−1^ and then converted in μmol O_2_ cm^−2^ h^−1^_._ Photosynthesis to respiration ratio (*P*_g_:*R*) was calculated using the value of daylight hours equal to 12 to calculate an integrated *P*_g_:*R* ratio for a 24 h day as follows:

(2) $$P_{g}:R=\frac{P_{g}\times \text{hours of daylight}}{|R_{\text{dark}}|\times 24}$$

#### 2.2.5 Tissue and surface measurements

All fragments used to assess the coral photosynthesis and respiration rates were prepared and analyzed for their Symbiodiniaceae and chlorophyll contents. Protein measurements were performed on the same individual used during photosynthesis and respiration incubations for *A. tenuis* and *M. digitata* and from a new individual for *Porites* sp. Then, their skeleton's surface areas were measured. Coral tissue was extracted from the skeleton using an air pick in 20 mL filtered seawater and homogenized with a Potter tissue grinder. For symbiont density measurement, 2 mL of the slurry was sampled to count the number of Symbiodiniaceae (*n* count = 8) using a Neubauer's cell under a stereomicroscope. 10 mL subsamples were centrifuged at 5000*g* for 10 min, the supernatant was discarded, and the pellet containing the symbiont was re‐suspended in 10 mL of pure acetone to extract during 24 h at 4°C in darkness the chlorophyll *a* and *c*_2_. The solution was then centrifuged at 10,000*g* for 15 min and the supernatant was sampled to measure its absorbance at 630, 663, and 750 nm using a spectrophotometer (Evolution 201, Thermo Scientific). Chlorophyll *a* and *c*_2_ concentrations were calculated using the spectrophotometric equations for dinoflagellates of Jeffrey and Humphrey (1975). Chlorophyll *a* and *c*_2_ are given as total chlorophyll expressed in μg cm^−2^.

Protein content was quantified using a BCA assay kit (Uptima, Interchim). Total protein was extracted according to (Hoogenboom et al., 2010) by incubating each fragment in a sodium hydroxide solution (1 N) maintained in a water bath for 30 min at 90°C. Samples were then diluted by a factor of 15 before being transferred into 96‐well microplates and incubated with a dye reagent (Uptima Reagents, Interchim) for 30 min at 60°C. Bovine serum albumin (BSA, Interchim) was used as a protein standard with concentrations of 0, 50, 100, 200, 350, 500, 750, and 1000 μg ml^−1^. Samples and standards were homogenized for 30 s on a microplate shaker within the spectrophotometer (Biotek ELx808); absorbances were measured at 563 nm, and the protein contents were calculated according to the standard equation and expressed in mg cm^−2^.

The skeletal surface areas of samples were estimated using the paraffin wax‐dipping method with two wax dips (Naumann et al., 2009; Stimson & Kinzie, 1991) for branching species (i.e., *A. tenuis* and *M. digitata*) and the aluminum foil technique (Marsh, 1967) for the massive species *Porites* sp.

### 2.3 Symbiodiniaceae community analysis

#### 2.3.1 DNA extraction, PCR amplification, and sequencing

Fragments of corals (*n* = 2–6 per species, depending on origin, and pH treatment) were collected and stored at −20°C. Total coral holobiont DNA (i.e., Symbiodiniaceae, polyp, and associated microorganisms DNAs) was extracted using a 2% CTAB‐based protocol adapted from (Mieog et al., 2009). The quantity and quality of extracted DNA were checked using a NanoDrop 2000 spectrophotometer (Thermo Fisher Scientific). Extracted DNA was then diluted to a range of 30–70 ng μL^−1^ for PCR amplification. The Symbiodiniaceae nuclear DNA ribosomal internal transcribed spacer (ITS2) region was amplified with the forward primer ITS2‐DINO [5′‐TCGTCGGCAGCGTCAGATGTGTATAAGAGACAGGTGAATTGCAGAACTCCGTG‐3′] (Pochon et al., 2001) and reverse primer ITS2Rev2 [5′‐GTCTCGTGGGCTCGGAGATGTGTATAAGAGACAGCCTCCGCTTACTTATATGCTT‐3′] (Stat et al., 2009). The underlined segments represent Illumina adapter overhangs (Illumina). The PCRs were conducted in 25 μL reactions using 12.5 μL of AmpliTaq 360 Master Mix, 1 μL of each 10 μM primer mix, 1 μL of 360 GC Enhancer, 2 μL of DNA template, and DNAse‐free water to adjust the reaction volume. The amplification cycle was set and adjusted (Arif et al., 2014) as follows: 94°C for 15 min; 35 cycles each at 95°C for 30 s, 49°C for 1 min, and 72°C for 30 s; and a final extension at 72°C for 10 min. To check amplification success, 3 μL of each PCR product was run on a 1% agarose gel. The resulting amplicons were sequenced using the Illumina MiSeq platform (2 × 300 bp) (Australian Genome Research Facility). Returned demultiplexed FASTQ files were analyzed via the SymPortal analytical framework (Hume et al., 2019). The SymPortal framework predicts from raw sequences, ITS2 type profiles from specific sets of defining intragenomic ITS2 sequence variants (DIVs) based on genetically differentiated Symbiodiniaceae taxa. Quality control was assessed using MOTHUR 1.39.5 (Schloss et al., 2009), BLAST+ suite of executables (Camacho et al., 2009), and minimum entropy decomposition (MED; Eren et al., 2015) to predict Symbiodiniaceae taxa from the ITS2 marker. All raw sequence data are accessible under NCBI's BioProject (PRJNA956479).

### 2.4 Statistical analyses and data presentation

Statistical analyses were conducted, and figures were produced using RStudio (R Development Core Team, version 4.1.0, 2021), including the packages *ggplot2*, *ggpubr*, *car*, and *vegan*. Homogeneity and the normality of variance distributions were tested using respectively the Levene test and the Shapiro–Wilkinson test and graphically verified with Q‐Q plots. Statistical analyses were performed separately for each species as the three species we used are morphologically different. Each response variable was evaluated with separate linear mixed‐effects models using the package *lme4* (Bates et al., 2015). To evaluate differences in the main environmental parameters in the tanks during the experiment, i.e., pH, temperature, and nutrients measurements, the full model included pH as a fixed factor and tank as a random factor. To evaluate coral physiological responses, the full model included coral's origin (two levels: Bouraké and reference) and pH conditions (four levels: control, future, extreme, and variable) as fixed factors and tank as a random factor. The significance of fixed factors was evaluated with two‐way ANOVAs (type III) using Satterthwaite's method. Significant differences between fixed factors were determined with Tukey's post hoc pairwise comparisons using the package *emmeans*. In contrast, *F*_v_ */F*_m_ data did not meet the assumption of normality and they were compared using the non‐parametric two‐way Aligned Rank Transformed (ART) ANOVA (Type III) followed by a Bonferroni *p*‐levels adjusted post hoc using the package *ARTool*. Data were described as box plots using median values ±25th and 75th percentiles (box), minimum and maximum values (whiskers), and dots as outliers, otherwise specified. Differences in Symbiodiniaceae ITS2 profiles were analyzed on square‐root transformed data using three‐factorial permutational multivariate analysis of variance (PERMANOVA) with 999 permutations of residuals and based on Bray‐Curtis distances to test for differences between sites (two levels), pH conditions (four levels), and species (three levels).

## 3 RESULTS

### 3.1 Seawater parameters

**FIGURE 1:**
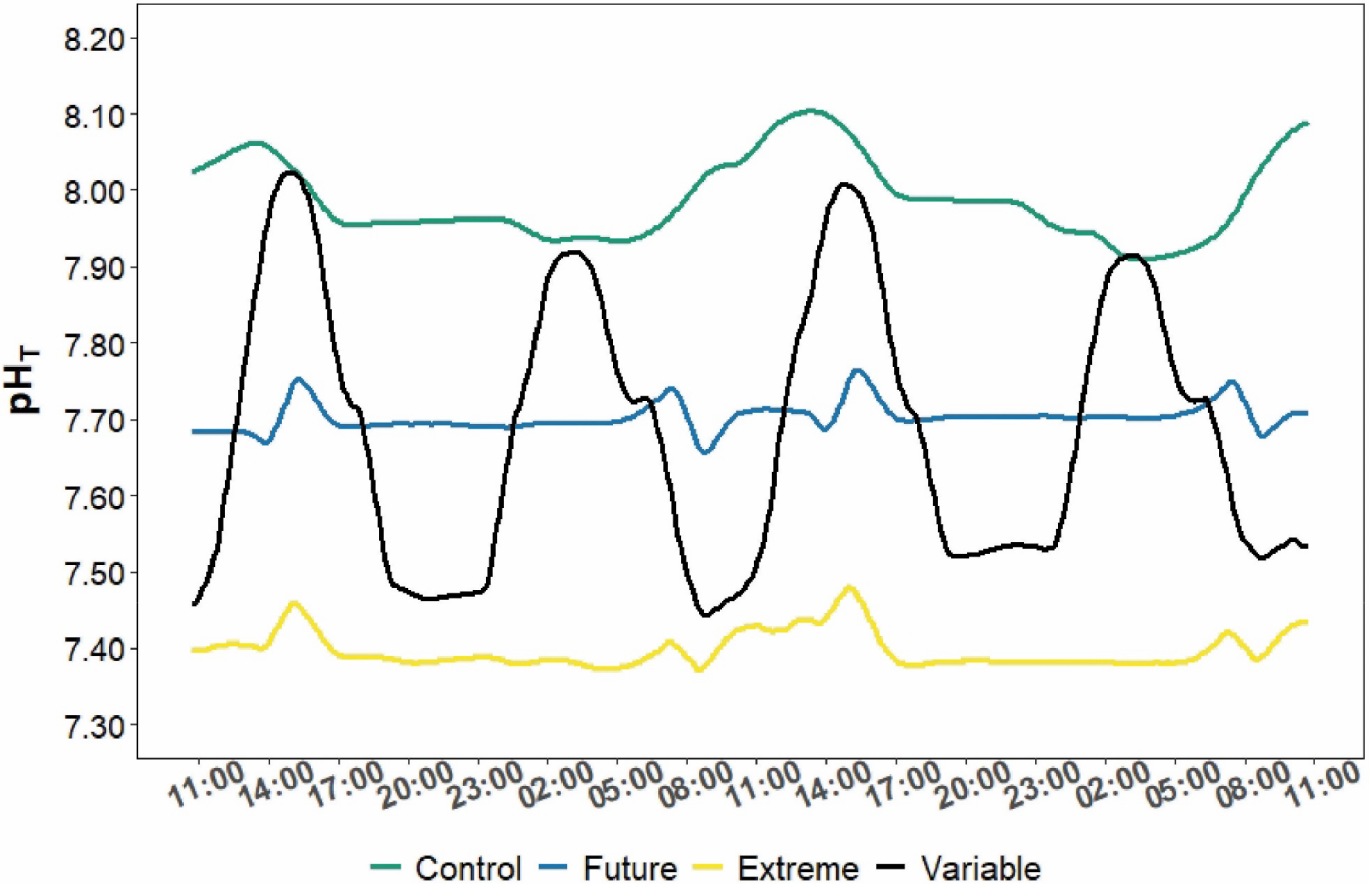
Seawater pH_T_ variations recorded with a SeaFet during a 48‐h cycle in the four monitored experimental pH conditions (Control; Future; Extreme; and Variable). See Table 1 and Table S3 for all data.

Seawater pH, temperature, and carbonate chemistry were maintained at the target experimental values during the 100‐day experiment (Table 1; Tables S3 and S4). There is a significant difference between conditions regarding temperatures (*p* < .001; Table S5). However, the average temperatures were rather similar between conditions (ranging from 26.18°C to 26.41°C). Minimum and maximum values were recorded in the extreme (23.90°C) and variable treatment tanks (28.30°C), respectively, due to a 24‐h malfunction of the temperature control system for this treatment that occurred during the first week of the experiment. The pH was significantly different (*p* < .001) between the four pH conditions with minimum and maximum values recorded in the extreme (7.31) and the control condition (8.27), respectively (Tables S3 and S5). According to the SeaFet measurements, seawater pH values varied by ca. 0.60–0.65 pH_T_ units in the variable condition, and by only 0.05–0.1 pH_T_ units in the other conditions (Figure 1).

Nutrient concentrations were similar between conditions (*p* > .620; Tables S5 and S6). Averaged values (±SD) of NO_x_ varied from 0.59 (±0.21) to 0.67 (±0.25) μmol L^−1^ for control and future conditions, respectively. PO_4_^3−^ concentrations were equal to 0.30–0.31 μmol L^−1^ for all treatments, while Si(OH)_4_ varied from 2.79 (±1.06) to 2.89 (±1.18) μmol L^−1^ for variable and future conditions, respectively.

### 3.2 Growth rate

**FIGURE 2:**
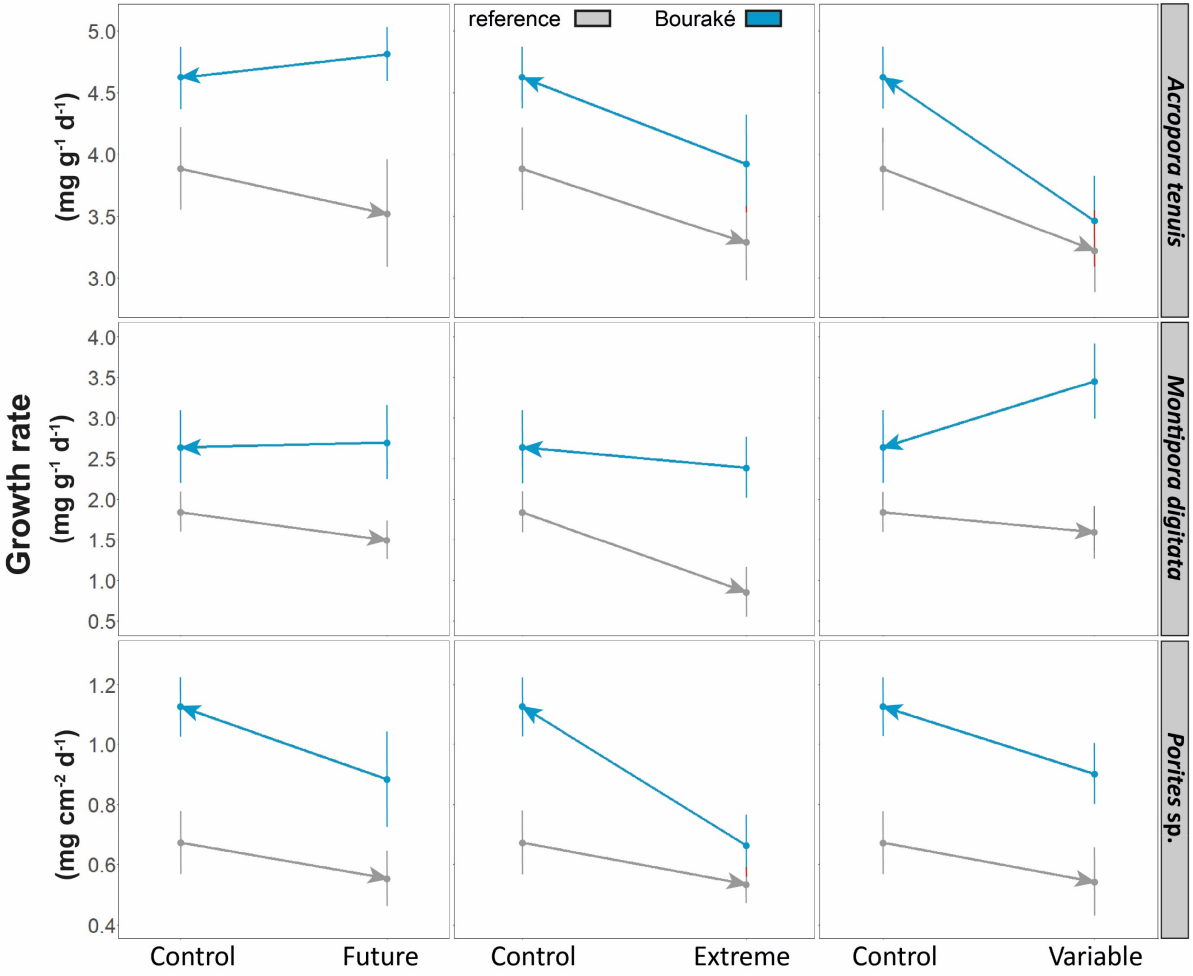
Reaction norm of the growth rates of corals from Bouraké (blue) and reference (gray) sites maintained during 100 days at four pH conditions (Control, pH_NBS_ 8.11; Future, pH_NBS_ 7.76; Extreme, pH_NBS_ 7.54; and Variable, pH_NBS_ 7.56–8.07). The arrow indicates the direction of the change from the condition of origin, assuming that corals from Bouraké might originate from seawater at either pH 7.7 (Future pH), 7.4 (Extreme pH), or fluctuating (Variable pH) and that corals from reference originate from seawater at pH 8.1 (Control pH). Data represented as dots are means of the three replicate tanks ± SE (overlapping SEs are in red) (*n* = 13–16, depending on species and pH condition; see Table S7 for all post hoc comparisons).

Growth rates of the three coral species (Figure 2) were significantly different according to corals' origin (*p* ≤ .003) and pH conditions (*p* ≤ .044), but not their interactions, with the exception of *M. digitata* showing a *p* = .092 for pH (two‐way ANOVA, Table 2 and Table S7 for all data analyses). The mean growth rates of the three corals originating from the reference and maintained at control pH, generally decreased when maintained at future, extreme, and also variable pH. This decrease was more evident in the two branching corals than in *Porites* sp. For instance, the calcification rates of *A. tenuis*, *M. digitata*, and *Porites* sp. originating from the reference site decreased by 9.3%, 19.0%, and 17.6%, respectively, in the future pH (pH_NBS_ 7.76) condition and by 15.4%, 53.5%, and 20.7% in the extreme pH (pH_NBS_ 7.54), compared to the control pH (pH_NBS_ 8.11).

**TABLE 2:** Two‐way ANOVAs (type III) summary with Satterthwaite's method of linear mixed effect model (LMER) on the effects of colony origin (O), pH conditions, and their interactions on the physiological responses of three coral species after 100 days of incubation.

Parameter	Fixed factor	*A. tenuis*	*M. digitata*	*Porites* sp.
Growth	Origin	0.003	<0.001	<0.001
	pH	0.020	0.092	0.044
	O × pH	0.491	0.519	0.473
*F*_v_/*F*_m_	Origin	0.001	0.717	0.274
	pH	0.018	0.606	0.559
	O × pH	0.803	0.180	0.881
rETR_max_	Origin	<0.001	0.858	0.822
	pH	0.379	0.445	0.586
	O × pH	0.939	0.457	0.615
*P*_g_	Origin	0.063	0.543	0.010
	pH	0.008	0.126	<0.001
	O × pH	0.297	0.096	0.052
*R*_dark_	Origin	0.022	0.717	0.009
	pH	0.002	0.163	0.018
	O × pH	0.316	0.468	0.372
*P*_g_:*R*	Origin	0.355	0.706	0.913
	pH	0.188	0.632	0.054
	O × pH	0.092	0.291	0.346
Symbiont	Origin	0.015	0.946	0.100
	pH	0.044	0.113	0.294
	O × pH	0.035	0.261	0.049
Total chl	Origin	0.002	0.802	0.576
	pH	0.058	0.770	0.210
	O × pH	0.278	0.462	0.032
Proteins	Origin	0.311	0.336	0.423
	pH	0.029	0.138	0.002
	O × pH	0.669	0.726	0.436

*Note*: Non‐parametric two‐way Aligned Rank Transformed (ART) ANOVAs (Type III) followed by a Bonferroni *p*‐levels adjusted post hoc test was used for *F*_v_ */F*_m_. Significant values are in bold (*p* < .05). All statistical analyses with the post hoc and random effects are in Table S7.

In contrast, the mean growth rates of the three corals originating from Bouraké and maintained at future, extreme, and variable pH (i) were always higher than their counterpart originating from the reference and maintained at the same pH condition (*p* ≤ .003); (ii) were almost always (with one exception, *A. tenuis* at variable pH) higher than their counterpart originating from the reference and maintained at the control pH condition; (iii) were almost always higher or did not change (with one exception, *M. digitata* at variable pH) when maintained at the control pH condition (Figure 2). For instance, the calcification rate of *A. tenuis*, *Porites* sp., *M. digitata* originating from Bouraké and maintained at the variable pH, which is what they normally experience at their site of origin, increased by 33.4% and 25.0% and decreased by 23.5%, respectively, in the control pH (pH_NBS_ 8.11).

### 3.3 Photosynthetic efficiency and electron transport rate

**FIGURE 3:**
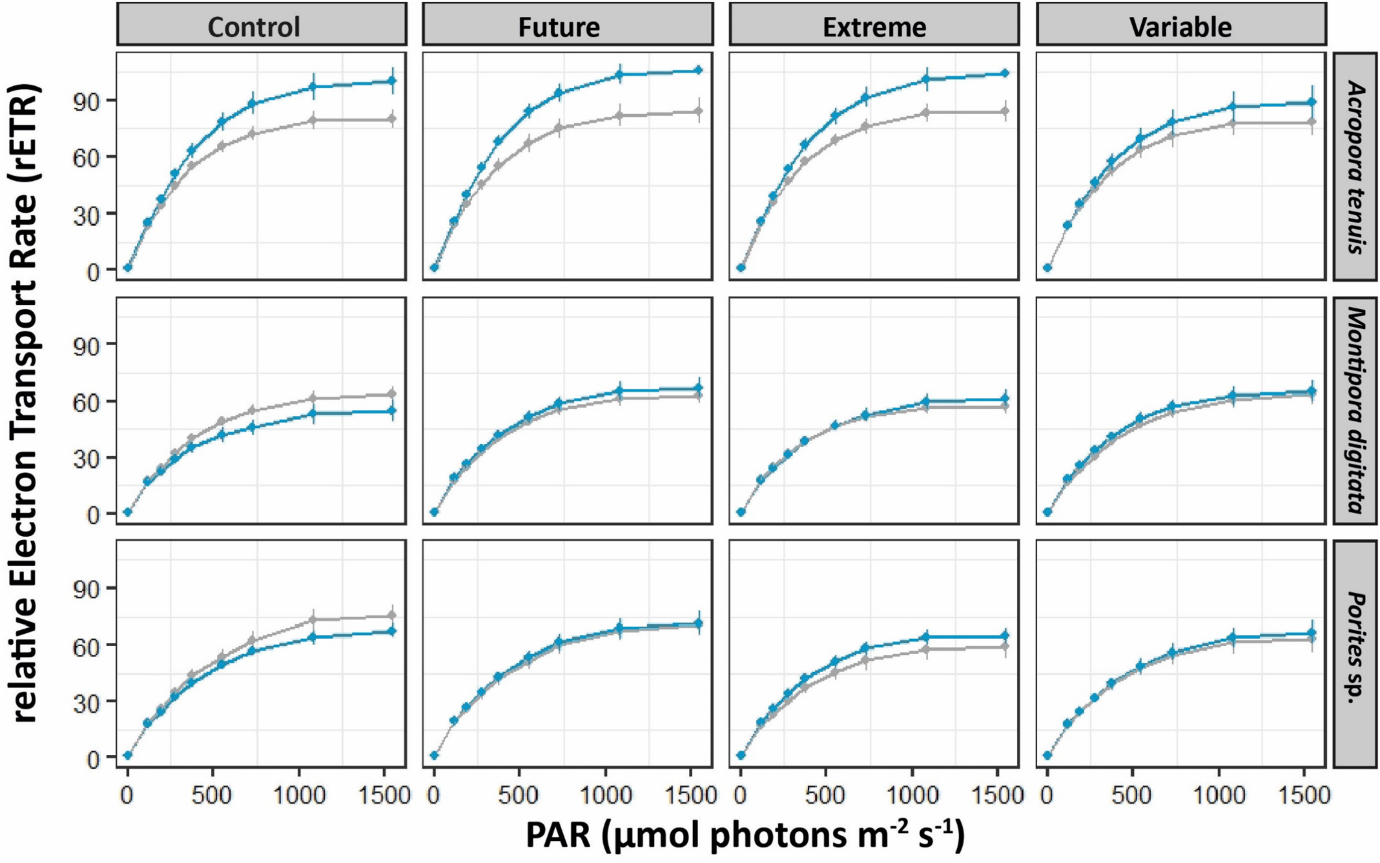
Relative electron transport rate (rETR) measured during RLC curves of corals after 100 days of incubation at four pH conditions (Control, pH_NBS_ 8.11; Future, pH_NBS_ 7.76; Extreme, pH_NBS_ 7.54; and Variable, pH_NBS_ 7.56–8.07). Bouraké corals are in blue, while reference corals are in gray. Data are mean ± SE; *n* = 12–16, depending on species and pH condition; see Table S7 for all post hoc comparisons.

The *F*_v_ */F*_m_ (i.e., maximum photochemical efficiency) and the rETR_max_ (i.e., maximum relative electron transport rates) were significantly different for *A. tenuis* only (Table 2; Table S7). The *F*_v_ */F*_m_ of *A. tenuis* differed according to both corals' origin (*p* = .001) and pH conditions (*p* = .018), whereas only corals' origin had an influence on rETR_max_ (*p* < .001). Both rETR_max_ (Figure 3) and *F*_v_ */F*_m_ (Figure S4) were higher for *A. tenuis* individuals from Bouraké, as compared to individuals from the reference site, regardless of the pH treatment. The *F*_v_ */F*_m_ for *A. tenuis* also showed higher values for future and extreme pH conditions than for control and variable pH conditions.

### 3.4 Photosynthesis and respiration rates

**FIGURE 4:**
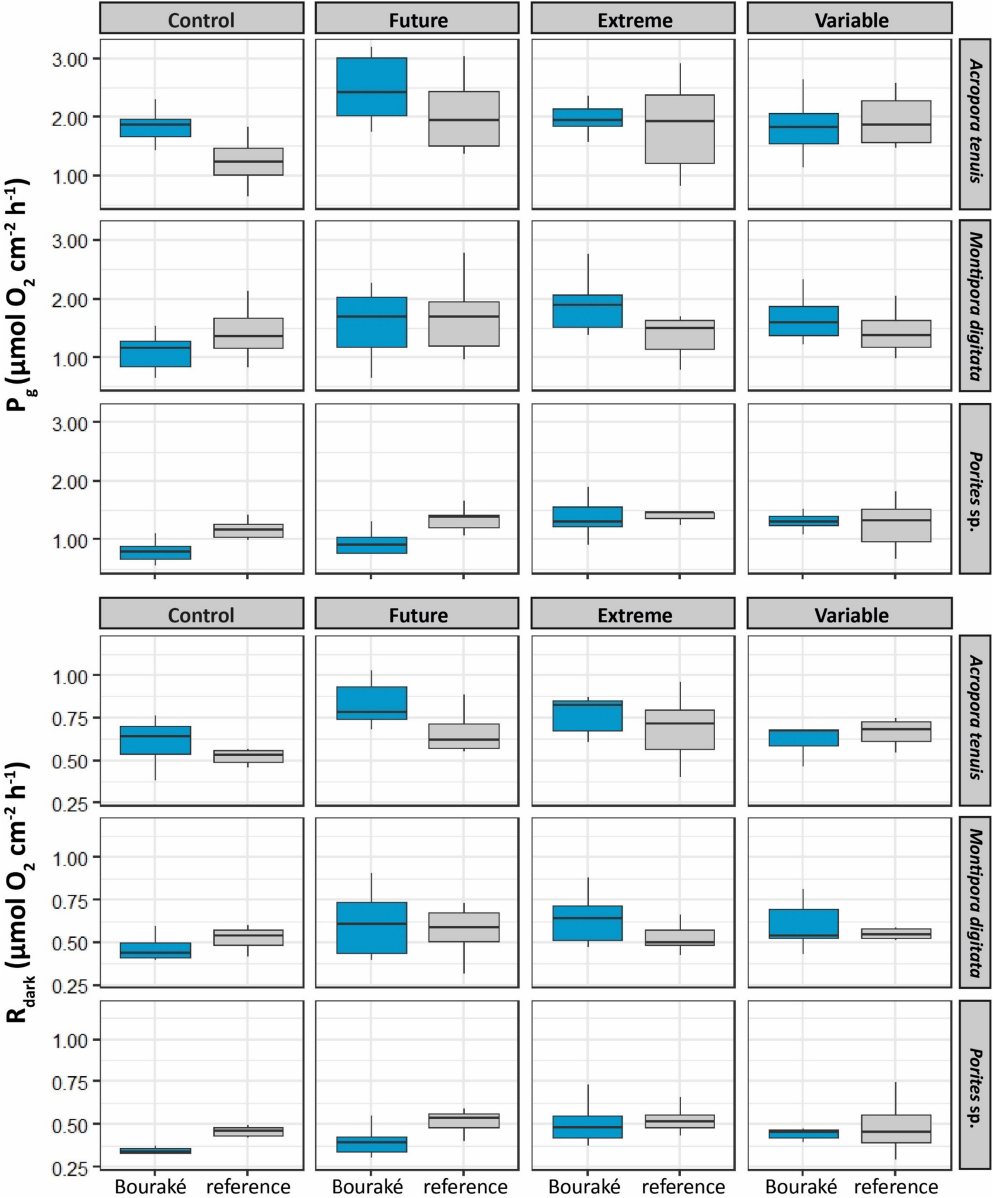
Gross photosynthesis (*P*_g_) and respiration (*R*_dark_) rates of corals from Bouraké (in blue) and the reference (in gray) site at four pH conditions (Control, pH_NBS_ 8.11; Future, pH_NBS_ 7.76; Extreme, pH_NBS_ 7.54; and Variable, pH_NBS_ 7.56–8.07). Data are median ± 25th and 75th percentiles (*n* = 7); see Table S7 for all post hoc comparisons.

Metabolic rates (i.e., *P*_g_, *R*_dark_, and *P*_g_:*R*) of the three coral species significantly varied between origins and pH conditions without a clear pattern of response. ANOVAs showed significant differences between origins in the *P*_g_ (only *Porites* sp., *p* = .010), *R*_dark_ (both *A. tenuis*, *p* = .022 and *Porites* sp., *p* = .009), but not for their ratio (*p* ≥ .355; Table 2; Figure S5; Table S7), and between pH conditions in the *P*_g_ (*p* ≤ .008) and *R*_dark_ (*p* ≤ .018) for *A. tenuis* and *Porites* sp. Overall, *P*_g_ and *R*_dark_ values were higher for future and extreme pH conditions for *A. tenuis* and *Porites* sp. (Figure 4; Table S7).

### 3.5 Symbiodiniaceae, chlorophyll and protein content

**FIGURE 5:**
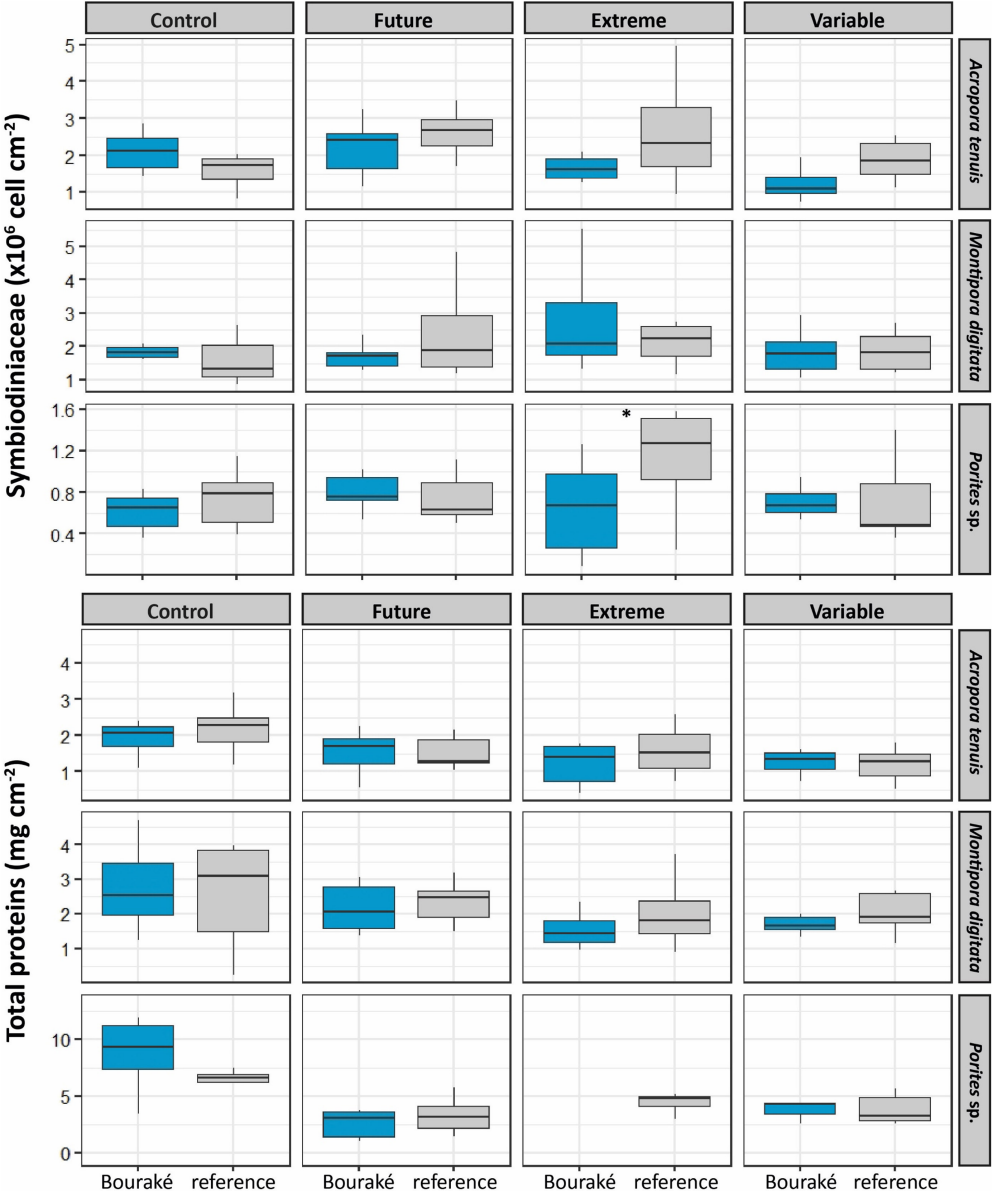
Symbiodiniaceae density and total protein content of corals from Bouraké (in blue) and the reference (in gray) site after 100 days of incubation at four pH conditions (Control, pH_NBS_ 8.11; Future, pH_NBS_ 7.76; Extreme, pH_NBS_ 7.54; and Variable, pH_NBS_ 7.56–8.07). Data are median ± 25th and 75th percentiles (*n* = 6–8, depending on species and pH condition). No data are available for the protein content of *Porites* sp. from Bouraké incubated at the Extreme pH condition. Asterisks indicate statistical significance at *p* < .05; see Table S7 for all post hoc comparisons.

A significant effect of pH (*p* = .044) was found only for Symbiodiniaceae density of the coral *A. tenuis* with higher density in the future condition and lower density in the variable condition (*p* < .050; Table 2; Table S7). Site of origin significantly affected the Symbiodiniaceae density and total chlorophyll content of *A. tenuis* (*p* ≤ .015; Table 2; Figure 5; Figure S6). Some interactions between pH and origin were also significant for *A. tenuis* (*p* = .035) regarding Symbiodiniaceae density and for *Porites* sp. regarding both Symbiodiniaceae density (*p* = .049) and total chlorophyll (*p* = .032). Most of these differences were driven by the higher data variability in extreme condition, especially for corals originating from the reference site (Figure 5; Figure S6; Table S7). Indeed, the post hoc comparisons highlighted the higher Symbiodiniaceae density found for individuals from reference in the extreme condition when compared to individuals from Bouraké in the variable condition for *A. tenuis* and in the extreme condition for *Porites* sp. (*p* < .050; Table S7). For protein, both *A. tenuis* and *Porites* sp. significantly differed between pH conditions (*p* = .029; Table 2; Table S6), and although values were very similar (Figure 5) post hoc comparisons showed higher protein contents for corals kept at the control pH condition compared to the variable (*A. tenuis*, *p* < .040) and future (*Porites* sp., *p* < .010) pH conditions.

### 3.6 Symbiodiniaceae communities

**FIGURE 6:**
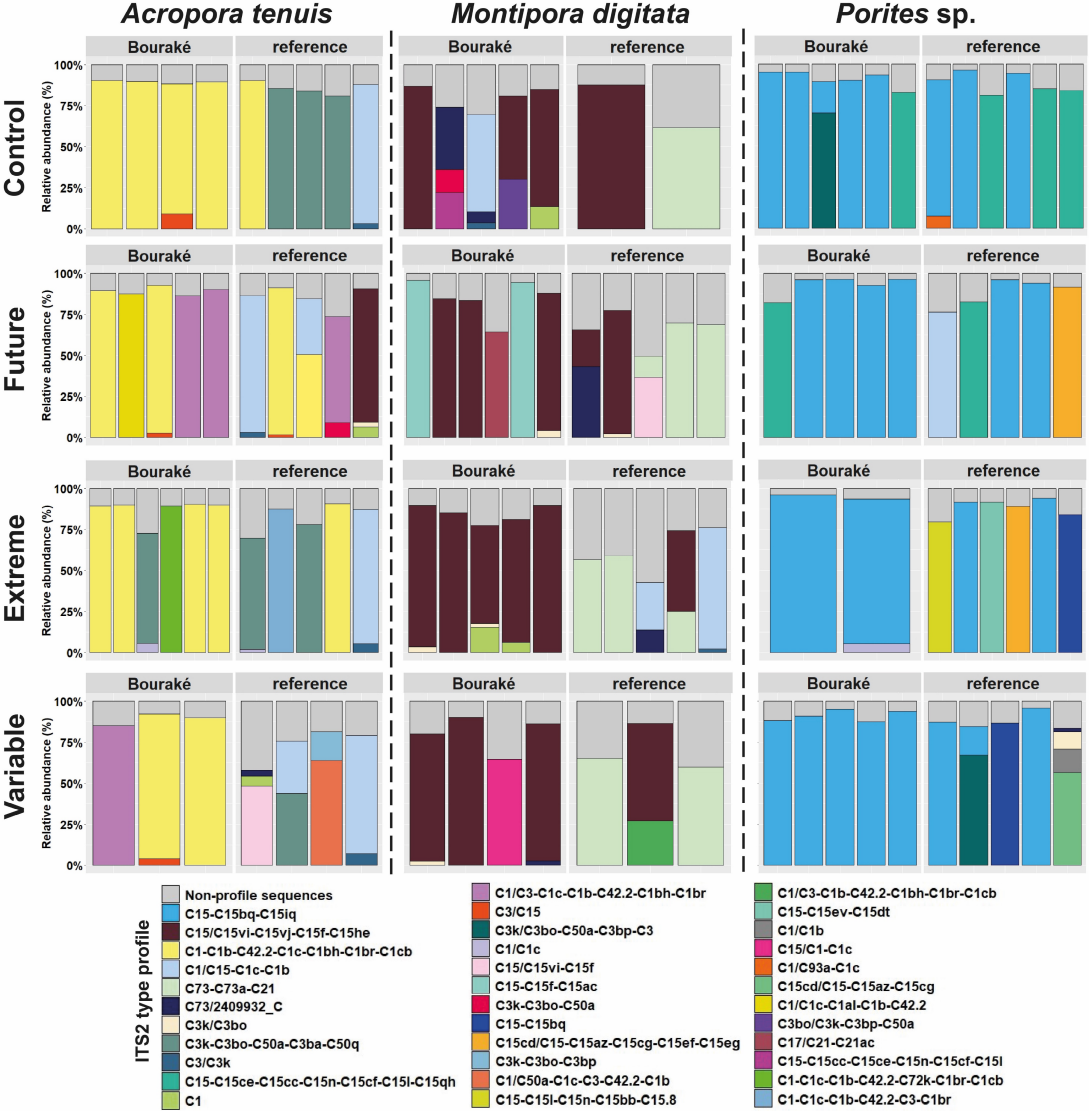
Predicted major ITS2 type profiles of corals from Bouraké and the reference site after 100 days of incubation at four pH conditions (Control, pH_NBS_ 8.11; Future, pH_NBS_ 7.76; Extreme, pH_NBS_ 7.54; and Variable, pH_NBS_ 7.56–8.07). Within each cell of this 3‐by‐4 matrix, samples are plotted as stacked bar charts with a single column representing a sample from a specific origin: Bouraké (left) and reference (right). The number of individuals per condition varies from 2 to 6 depending on the number of samples available.

The three coral species were consistently associated with Symbiodiniaceae of the genus *Cladocopium* but were dominated by distinct ITS2 type profiles. The latter were significantly different between species, coral origin, and their interaction (*p* < .001; Table 3; Figure 6). The major ITS2 type profiles (most abundant) of corals from the reference site comprised 58.1%–91.1%, 42.6%–87.5%, and 76.4%–96.2% of the total sequences in each sample for *A. tenuis, M. digitata, and Porites* sp., respectively. Under control conditions, *A. tenuis* had 4 major ITS2 DIV type profiles, with C3k‐C3bo‐C50a‐C3ba‐C50q most common (3 of 5 samples), while *Porites* sp. had 3 major type profiles, with C15‐C15ce‐C15cc‐C15n‐C15cf‐C15l‐C15qh most common (3 of 5 samples). Two major type profiles were found in *M. digitata* (C15/C15vi‐C15vj‐C15f‐C15he and C73‐C73a‐C21). For corals from Bouraké, the major ITS2 type profiles comprised 72.6%–92.5%, 64.5%–95.7%, and 82.2%–93.3% of the total sequences in each sample for *A. tenuis, M. digitata, and Porites* sp., respectively. For *A. tenuis*, the major type profile was C1‐C1b‐C42.2‐C1c‐C1bh‐C1br‐C1cb (one sample was also C3/C15), while *Porites* sp. was predominantly C15‐C15bq‐C15iq (one replicate was C15‐C15ce‐C15cc‐C15n‐C15cf‐C15l‐C15qh). *M. digitata* had 9 major type profiles, with C15/C15vi‐C15vj‐C15f‐C15he the most common (found in 50% of the samples).

**TABLE 3:** Three‐factorial PERMANOVA analysis testing the effects of colony origin (O), pH conditions, coral species, and their interactions on the Symbiodiniaceae ITS2 type profiles.

Parameter	*df*	*F*‐value	*p*‐value
Origin	1	5.109	**<.001**
pH	3	1.141	.275
Species	2	18.736	**<.001**
O × pH	3	0.882	.649
O × Species	2	5.526	**<.001**
pH × Species	6	0.894	.688
Origin × Species	6	0.733	.937
Residuals	88		

*Note*: Significant values are in bold (*p* < .05).

The pH treatments did not result in a significant change in the Symbiodiniaceae major type profiles for any coral species (*p* = .275; Table 3). However, an interesting observation was that in general corals from Bouraké had more consistent major ITS2‐type profiles between replicate colonies, than corals from the reference site (Figure 6). Individuals of the three coral species originating from the reference site had three times more type profiles when maintained at future or variable pH conditions than individuals under the control pH.

## 4 DISCUSSION

Corals from the Bouraké lagoon have evolved over generational time scales in an environmental condition (pH, temperature, and dissolved oxygen) chronically exceeding those predicted by the IPCC scenarios for the end of this century (IPCC Report, 2021). The Bouraké geomorphology, coupled with an intense oxidative activity in the mangrove sediments, has been shown to cause seawater acidification, deoxygenation, and warming in the lagoon (Maggioni et al., 2021). Despite the extreme levels of acidification measured, which have been suggested to hinder coral calcification (Kleypas et al., 1999), an abundant, and well‐diversified coral reef has developed in the lagoon. By assessing the holobiont physiological responses and the Symbiodiniaceae profiles of three coral species from both Bouraké and a reference reef to a large range in pH, we found that corals from Bouraké always exhibited higher growth rates and had a specific and more consistent ITS2 majority sequence than corals from the reference reef both under low and variable pH conditions. It seems likely that such patterns were linked to the strong life‐long environmental fluctuations, which might have promoted coral resilience as previously suggested for other corals (e.g., Brown et al., 2022; Comeau et al., 2021; Enochs et al., 2020; Rivest et al., 2017; Schoepf et al., 2020). We are aware that this study explored only a few of the compensatory mechanisms that might be at the origin of the resilience observed for corals from Bouraké. We also admit that these coral populations may have developed such mechanisms in a much longer time scale than corals that will have to cope with rapid climate change. However, results from this study would suggest that adaptation to future OA conditions could be possible in the wild.

### 4.1 Coral potential resilience to ocean acidification: What do we learn from lifetime adapted corals?

In agreement with the consensus on the effect of OA on coral calcification (Gattuso et al., 1999), recently revised by Leung et al. (2022), we found that calcification rates of the both *A. tenuis* and *Porites* sp. reference corals were significantly decreased when exposed to future and extreme pH levels. These results are consistent with coral species‐specific responses to OA with respect to calcification. We observed species‐specific responses to OA on corals from Bouraké although almost always (with one exception, *A. tenuis* at variable pH) showed higher calcification rates than their counterpart originating from the reference and maintained at the control pH condition. The marginal effect of OA observed in corals from Bouraké has been observed on some other coral species (e.g., *Pocillopora damicornis*; *Porites cylindrica; Siderastrea siderea*) in previous aquaria experiments (Aichelman et al., 2021; Bell et al., 2022; Brown et al., 2022; Comeau et al., 2013, 2019) and has recently been the subject of meta‐analysis and reviews (Bove et al., 2020; Leung et al., 2022).

Our data showed that all three coral species from Bouraké calcified from 19.0% (*A. tenuis*) to 67.3% (*Porites* sp.) more than their counterpart from the reference site, when maintained at control pH (Figure 2a,b). This unexpected finding suggests that Bouraké corals have adapted to OA because they have been exposed to extreme conditions throughout their lives, and regain even higher rates of calcification than the same coral counterparts adapted to open‐water pH conditions, once at actual open‐water pH (i.e., ca. 8.10).

The innovative aspect of our study is that we compared corals adapted to ambient seawater pH with corals likely adapted, or at least fully acclimated, to an extreme, and fluctuating environment overlaying future climate scenarios. This study demonstrated that Bouraké corals have become more resilient to OA, likely using plastic and/or assimilated mechanisms such as changes in coral‐associated microorganisms (Camp et al., 2020). Brown et al. (2022) incubated in aquaria with stable (218 ± 9 μatm) and variable daily *p*CO_2_ amplitude (911 ± 31 μatm) conditions the coral *Pocillopora damicornis* from both a flat and a sloping reef, where mean daily *p*CO_2_ amplitude differed (797 ± 20 and 399 ± 8 μatm d^−1^, respectively). As in our study, they found higher rates of calcification for corals from the more variable environment. The authors measured lower secondary calcification (i.e., CaCO_3_ density) on corals from the variable environment with less intracellular pH acidosis, as previously found for other corals (Comeau et al., 2021; Cornwall et al., 2018; Gibbin & Davy, 2014). Clearly, this compensatory mechanism might have a cost. Among the potential hypotheses explaining how corals can cope with the additional energy required to maintain high calcification rates using for instance proton pumping (Guillermic et al., 2021; McCulloch et al., 2012), it is known that corals: (i) might boost their endosymbiotic algal production (Barott et al., 2015; Castillo et al., 2014; Schoepf et al., 2013), (ii) use their energy reserves, such as proteins and lipids (Edmunds & Wall, 2014; Towle et al., 2015), and (iii) increase heterotrophy (Edmunds, 2011; Houlbrèque et al., 2015).

Our data do not consistently suggest that Bouraké corals have acquired a particular mechanism that accounts for better physiological plasticity to cope with low pH conditions, thereby maintaining higher calcification rates. Indeed, we did not find a clear effect of coral origin with a systematic increase in photosynthetic rates, higher contents of Symbiodiniaceae, chlorophylls, and protein in Bouraké corals. However, our study confirms previous findings on the positive effect of high *p*CO_2_ on photosynthetic rates, which might boost endosymbiotic photosynthates production for the coral proton pumping mechanism. It is well known that under acidified conditions at the cellular level, the photosynthetic activity of algal symbionts in coral increases until it exceeds the maximum level of CO_2_ consumed by the algae (e.g., Barott et al., 2015; Gattuso et al., 1999; Gibbin & Davy, 2014). Although CO_2_‐induced photosynthetic fertilization of symbionts may be less effective for *M. digitata*, *A. tenuis,* and *Porites* sp. showed higher photosynthesis under OA scenarios compared to the control condition. This was accompanied by higher ETR_max_, *F*_v_ */F*_m_ values, and better exploitation of lower light intensities for *A. tenuis*. This species also had the highest growth rates, which highlights the symbionts involvement in coral growth. In our study, we suggest that higher productivity was supported by an increased concentration in the Symbiodiniaceae density and/or chlorophylls, but not consistently among species highlighting the variable effect of OA on productivity such as previously reported (Anthony et al., 2008; Wall et al., 2014). Moreover, the respiration rates were higher under acidified conditions for *A. tenuis* and *Porites* sp. The high photosynthetic rates are consistent with the beneficial effect of elevated *p*CO_2_ on the productivity of corals living in CO_2_ vents in Papua New Guinea (Biscéré et al., 2019). These trends of increasing photosynthetic and respiration rates under OA do not match with the observations made by Jacquemont et al. (2022), for whom OA had no effect on photosynthetic and respiration rates of Bouraké corals, although, unlike our study, they found significant different rates between Bouraké and the reference site. Differences between the two studies could be due to the different setup of the two experiments as Jacquemont et al. (2022) measured coral metabolic rates under conditions mimicking the Bouraké environment, at high and low tide, and using seawater collected directly during both tidal periods. We measured the effect of medium‐term incubation at different seawater pH on corals from Bouraké and the reference site, while Jacquemont et al. (2022) tested the effect of a combination of factors, including pH, oxygen, and organic matter.

This study also found significant depletion of protein levels in all pH treatments, although this loss remained equivalent between the two corals origins, suggesting an alternative energy source used by Bouraké corals to maintain higher growth rates. Interestingly, *A. tenuis* and *Porites* sp. had lower protein levels under variable pH, suggesting that corals must bear an additional cost to cope with the large change in pH measured at Bouraké. The negative effect of OA on coral protein metabolism could be the result of accelerated protein catabolism at elevated *p*CO_2_ (Edmunds & Wall, 2014), which can be exacerbated when pH fluctuates over time. The loss of proteins under acidified conditions could also be explained by the species‐specific ability of corals to preferentially allocate energy either toward inorganic growth (calcification) or somatic growth (tissues) when facing elevated *p*CO_2_ (Agostini et al., 2021). We recognize that protein content can only partially describe the change in the coral energy reserves, since lipids and carbohydrates were not measured. Corals exposed to higher *p*CO_2_ might catabolize energy reserves to increase or maintain calcification, although this response is species‐specific (Grottoli et al., 2018; Schoepf et al., 2013; Wall et al., 2017). In our study corals were fed once a week with *Artemia salina* nauplii and such heterotrophic inputs might have helped them (Cohen & Holcomb, 2009; Drenkard et al., 2013; Edmunds, 2011; Houlbrèque et al., 2015). Indeed, it has been observed that the artificial diet we used, although limited compared to previous studies, helps corals maintain appropriate energy expenditure and calcify under acidified conditions (Houlbrèque et al., 2015; Houlbrèque & Ferrier‐Pagès, 2009). This is one of the potential limitations of most existing experiments in aquaria. Indeed, it is difficult to imitate the natural contribution of zooplankton to the diet of corals in an aquarium. However, all individuals were fed in the same manner and the nutrient levels (i.e., NO_x_, PO_4_^3−^, and Si(OH)_4_) measured in the aquaria were quite similar between tanks and throughout the experiment. Thus, the diet could perhaps explain some of the apparent resistance of corals to OA, but not the different responses with regard to calcification between Bouraké and the reference site.

### 4.2 To what extent do pH fluctuations improve the physiological performances of corals?

One of our hypotheses to explain the success of Bouraké corals was the potential positive effect that diurnal pH fluctuations could have on coral metabolism, as previously found for corals in GBR mangrove lagoons (Camp et al., 2019), St Vincent and the Grenadines CO_2_ vents (Enochs et al., 2020), and for others incubated in mesocosms (Brown et al., 2022; Dufault et al., 2012). Our results show that short‐term exposure (i.e. acclimation of reference corals during our experiment) to fluctuating pH has a negative effect on coral calcification as all coral species from the reference decreased their growth when incubated at variable pH (Figure 2c). In contrast, Bouraké corals, which are acclimatized and/or adapted to local conditions, when exposed to variable pH calcified from 7.6% (*A. tenuis*) to 116.2% (*M. digitata*) more than their counterpart from the reference site. Furthermore, Bouraké corals incubated at future and extreme pH maintained these higher growth rates and even increased them when grown under control conditions, confirming their resistance to OA. Although the duration of our experiment was longer than most OA experiments (i.e., 77% lasted 1–11 weeks; Brown et al., 2022; Ziegler et al., 2021), we acknowledge that the duration of variable pH exposure experienced by corals from the reference site during the course of the experiment was too short to compare with that experienced by corals at Bouraké. Corals exposed to a variable environment throughout their life are physiologically more plastic than corals adapted to stable environments (Kenkel & Matz, 2017); such plasticity is probably time‐dependent. Future experiments should take into account the length of exposure to variable pH (as well as other environmental parameters) that the corals experienced before the collection.

### 4.3 Does their Symbiodiniaceae community raise the physiology of Bouraké corals?

It is thought that species‐specific metabolic responses to environmental stress could be due to different symbiont communities hosted by corals (Barott et al., 2015; Ziegler et al., 2015). Our data suggest that seawater pH level, whether stable or variable, at future or extreme levels, does not affect the Symbiodiniaceae major ITS2‐type profiles of corals (at least over 100 days), since at the end of the experiment we found no significant effect among treatments. Interestingly, however, Bouraké corals exhibited more consistent major ITS2‐type profiles between replicate colonies at low and variable pH than corals from the reference site. Microbiome stability (both for symbionts and/or bacteria) was linked to greater physiological resilience to OA (Ge et al., 2021; Grottoli et al., 2018; Quigley et al., 2017, 2019; Ros et al., 2021), suggesting that consistent major ITS2‐type profiles for Bouraké corals under pH treatments could facilitate their success; however, further work will be needed to verify this hypothesis.

We observed distinct major ITS2‐type profiles between coral species and native habitat (e.g. Bouraké versus reference site). All coral species in this study were associated with the genus *Cladocopium* (LaJeunesse et al., 2018). We could have expected to find the Symbiodiniaceae genus *Durusdinium* as it is often found in stress‐tolerant corals (Haydon et al., 2021; Hoadley et al., 2019; Lajeunesse et al., 2014). However, some species of *Cladocopium* seem to be competitively dominant against *Durusdinium* in a multi stressors environment such as Bouraké (Barshis et al., 2010; Hume et al., 2016). For example, *Cladocopium* was found to be the dominant genus on corals species such as *Porites lutea* from the mangrove lagoon of Woody Isles (Australia, Camp et al., 2019), and both *Acropora pulchra* and *A. muricata* in Bouraké (Camp et al., 2020). In Bouraké, distinct symbiont‐type profiles for each coral host species, across environments, support previous hypotheses of species‐specific strategies of environmental adaptation (Camp et al., 2020):

(i) *A. tenuis* in Bouraké had a major type profile of C1‐C1b‐C42.2‐C1c‐C1bh‐C1br‐C1cb. Corals associated with C1 as a major ITS2 sequence have been found across many environments (LaJeunesse et al., 2003), and exhibit apparent capabilities to adapt to locally stressful and/or fluctuating environments (Howells et al., 2012; Ng & Ang, 2016; Schoepf et al., 2015). Corals from Bouraké exhibited lower Symbiodiniaceae density than the reference corals but had higher photosynthetic efficiency. Corals associated with *Cladocopium goreaui* (C1) have previously shown elevated photosynthetic efficiency with regard to other Symbiodiniaceae species (Cantin et al., 2009; Morgans et al., 2020; Stat, Morris, & Gates, 2008; Wall et al., 2020). It is therefore possible that elevated photosynthesis is a common trait within some taxa from the C1 radiation and may explain the higher photosynthetic efficiency mechanism of C1 to face OA as observed by Ge et al. (2021) in *Acropora valida*. The elevated photosynthetic capacity of *A. tenuis* in Bouraké, coupled with the dominance of major ITS2 sequence C1, could explain their significantly higher growth rates, as previously observed in *A. tenuis* juveniles (Cantin et al., 2009; Little et al., 2004). The C1 ITS2 sequence was still dominant for some colonies of *A. tenuis* from the reference site (albeit different ITS2 type profiles that suggest different species), but in most instances, ITS2 sequences from the C3 radiation (i.e., C3k and C3bo) were most abundant; these are not among the most efficient in photosynthetic capabilities (Hoadley et al., 2016). We note the uncommon prevalence of C15 in *A. tenuis* (Figure S7). We are confident from our negative control gels that this is real (Figure S8), and could perhaps result from expelled symbionts from the other coral taxa during the stress experiment. Further work would need to validate this hypothesis.

(ii) *Porites* sp. from both Bouraké and the reference site maintained an association with *Cladocopium* of the C15 radiation, but there were more numerous C15 major ITS2 type profiles at the reference site. Interestingly, discrete C15 genotypes (distinct ITS2 type profiles) were observed across habitats (i.e., extreme vs. reference) in *P. lutea* from Bouraké (Camp et al., 2020) and from a mangrove lagoon on the Great Barrier Reef (Camp et al., 2019). This is in agreement with the recent finding of Hoadley et al. (2021) demonstrating that within C15, diverged lineages exist for *Porites* sp. living in different reef habitats and that it brings physiological differences.

*(iii) M. digitata* from Bouraké had nine major ITS2 type profiles, with C15/C15vi‐C15vj‐C15f‐C15he the most common. *Cladocopium* of the C15 radiation are considered physiologically resistant (Fisher et al., 2012; Fitt et al., 2009; Nitschke et al., 2018). The same C15 major type profile was also found for the reference corals, alongside C73‐C73a‐C21. Coral association with C73 is rarely found and appears to be present according to relative light levels (Stat, Loh, et al., 2008). With this in mind and according to the equivalent light irradiance measured between tanks in our experiments, we could assume that light regimes of the native environment of corals modified their Symbiodiniaceae community associations. For instance, turbid environments such as Bouraké have been observed to limit the diversity of the associated symbiont community and could lead to predominant local adaptive genotypes (Smith et al., 2020). While it is beyond the intent of this study to determine which environmental drivers shape symbiotic communities, our data supports the growing body of evidence (Camp et al., 2020; Howells et al., 2016; Ziegler et al., 2017) that corals from distinct environments often have unique symbiotic partners that could be crucial to support their survival.

While we cannot define the role of each Symbiodiniaceae ITS2‐profile in the coral stress response, the difference in symbiotic partners' genotypes between sites highlights how important local environmental conditions are and how symbiotic stability (fidelity) versus flexibility ultimately enhances holobiont resilience (Epstein et al., 2019; Grottoli et al., 2018; Putnam et al., 2012).

In conclusion, we show that under the OA scenarios tested here, corals adapted to an extreme and variable environment have systematically higher calcification rates than reef corals living at a more stable environment. Although not all physiological measurements revealed significant differences between coral's origin, Bouraké corals showed that their calcification rates have been somewhat boosted, regardless of pH level and variability. Concomitantly, they maintain divergent symbionts with more consistent Symbiodiniaceae communities than corals from a more stable environment. The comparison between corals that have been exposed during their life to variable pH with corals exposed only during the 100‐day incubation suggests that this is not rapid mechanism. Upcoming studies should explore the effects of the exposure duration (i.e., short vs. long‐term) to variable pH on coral biomineralization mechanisms, which already appear to differ between more stable and fluctuating environmental conditions of pH (Comeau et al., 2021). The different dynamics of symbiotic partners might greatly influence calcification processes via distinct translocation of photosynthates ultimately influencing mechanisms of internal pH regulation (Allen‐Waller & Barott, 2023; Cameron et al., 2022; Venn et al., 2022). The use of isotopic tools such as the boron pH proxy in the coral skeleton would help that way, via tracing and defining the inputs of specific symbiotic partners into biomineralization mechanisms.

## AUTHOR CONTRIBUTIONS

**Clément Tanvet:** Conceptualization (lead); data curation (lead); formal analysis (lead); investigation (lead); methodology (lead); writing – original draft (lead); writing – review and editing (lead). **Emma F. Camp:** Formal analysis (supporting); funding acquisition (supporting); investigation (supporting); methodology (supporting); writing – review and editing (supporting). **Jill Sutton:** Supervision (supporting); writing – review and editing (supporting). **Fanny Houlbréque:** Methodology (supporting); writing – review and editing (supporting). **Gérard Thouzeau:** Funding acquisition (lead); supervision (supporting); writing – review and editing (supporting). **Riccardo Rodolfo‐Metalpa:** Conceptualization (lead); data curation (supporting); formal analysis (supporting); funding acquisition (lead); investigation (lead); methodology (supporting); supervision (lead); writing – review and editing (supporting).

## FUNDING INFORMATION

CT received a PhD fellowship from the University of Western Brittany (UBO) and the Brittany region (France). This study was supported by the French grant scheme Fonds Pacifiques (no. 1976; project SuperCoraux), the CNRS (MITI interdisciplinary programs), ISblue (Interdisciplinary graduate school for the blue planet; ANR‐17‐EURE‐0015), and a grant from the French government under the program “Investissements d'Avenir”. Contribution of EFC to the project was supported by an ARC Discovery Early Career Research Award (DE190100142) and University of Technology Sydney Chancellor's Postdoctoral Research Fellowship awarded to EFC. This project contributes toward the International CO_2_ Natural Analogues (ICONA) Network.

## Supporting information


Appendix S1

## Data Availability

Additional supporting information may be found online in the Appendix S1 for this article. All data are available from the Dryad Digital Repository: https://doi.org/10.5061/dryad.zgmsbccg8. All raw sequence data as fastq read files are accessible under NCBI Sequence Read Archive (SRA), under NCBI's BioProject: PRJNA956479.
