# Supplementary material for: Corals adapted to extreme and fluctuating seawater pH increase calcification rates and have unique symbiont communities
Source: Ecol Evol. 2023 May 29;13(5):e10099. doi: 10.1002/ece3.10099 (PMC10227177; doi:10.1002/ece3.10099)
Supplement: Supplementary file 1 — Appendix S1 [file ECE3-13-e10099-s001.docx]

**Appendix A**


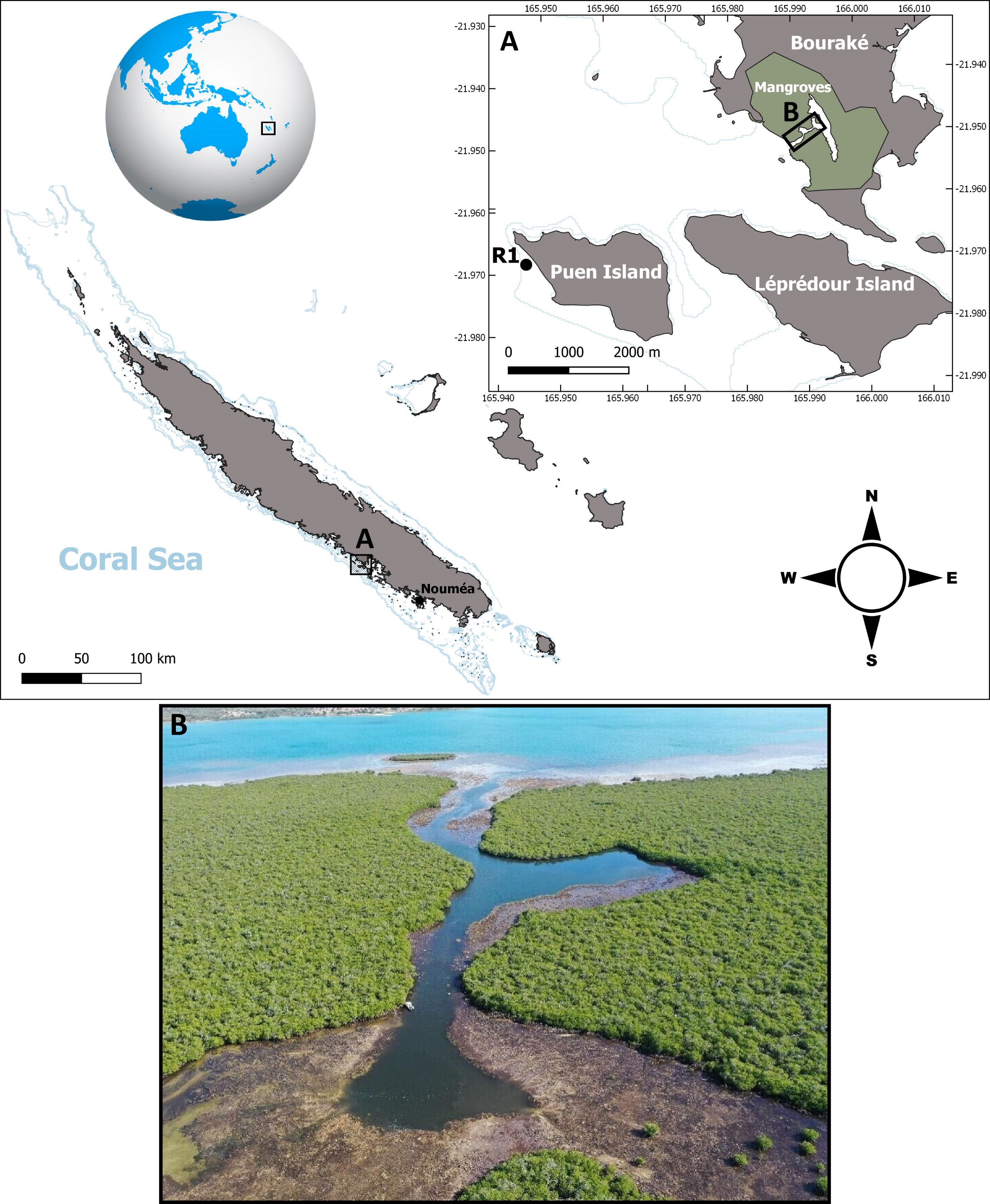


**Figure A1.** Map of the study sites (B and R1) in New Caledonia where corals were collected. Photo (B) is an aerial pic of the Bouraké lagoon (taken at 130 m height).

**
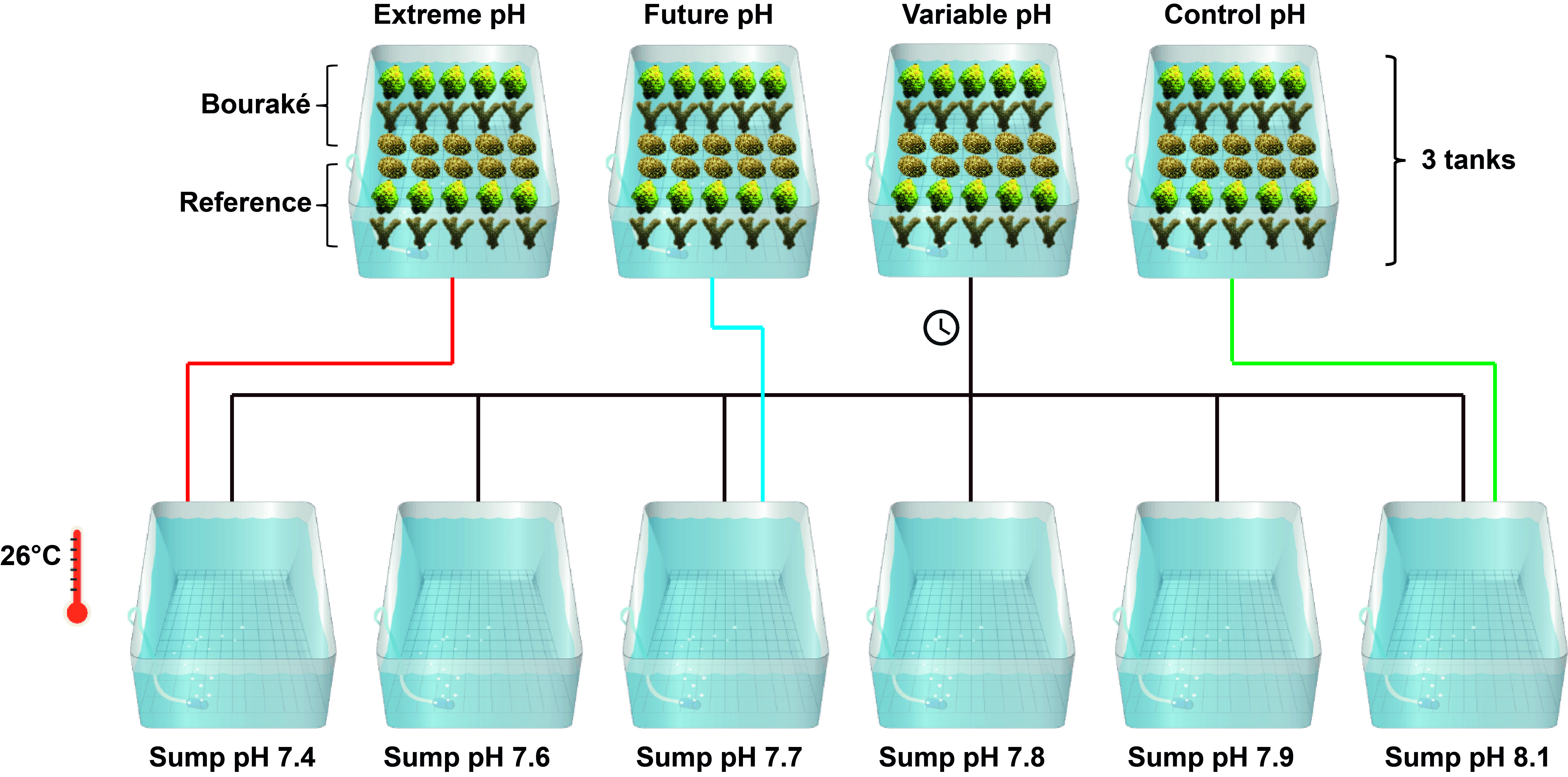
**

**Figure A2.** Schematic representation of the experimental set up with four pH conditions (Control, pH_NBS_ 8.11; Future, pH_NBS_ 7.76; Extreme, pH_NBS_ 7.54; and Variable, pH_NBS_ 7.56-8.07), each replicated in three experimental tanks, and 6 tank sumps that alimented the experimental tanks. In each experimental tank, 30 coral fragments were positioned, half from Bouraké and half from the reference site. The Variable condition was supplied by one or more sumps simultaneously according to a time table mimicking pH variation in Bouraké (see Table A2). Sump temperature was maintained at ca. ~26°C.

**
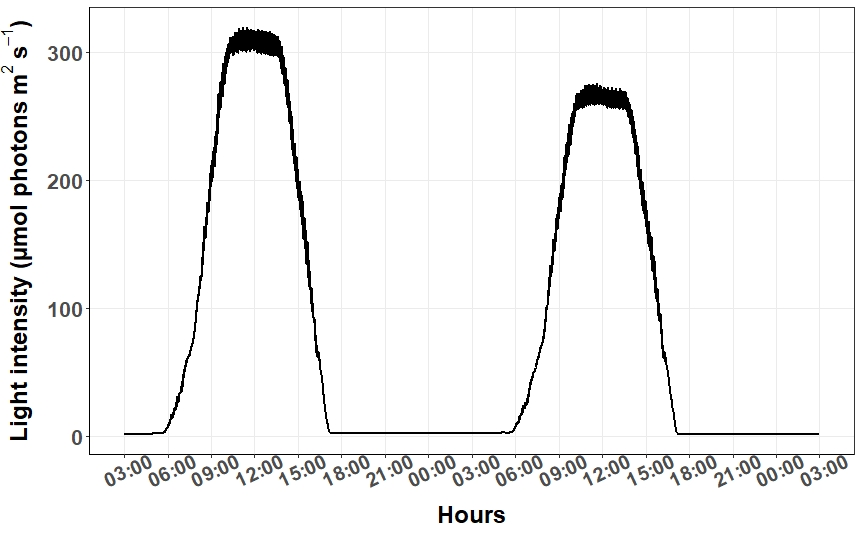
**

**Figure A3.** Example of the light intensity received by the corals during the 100-day incubation, and measured in two tanks having a different position below the LED lights (see Tables A1 and A2 for setting).

**
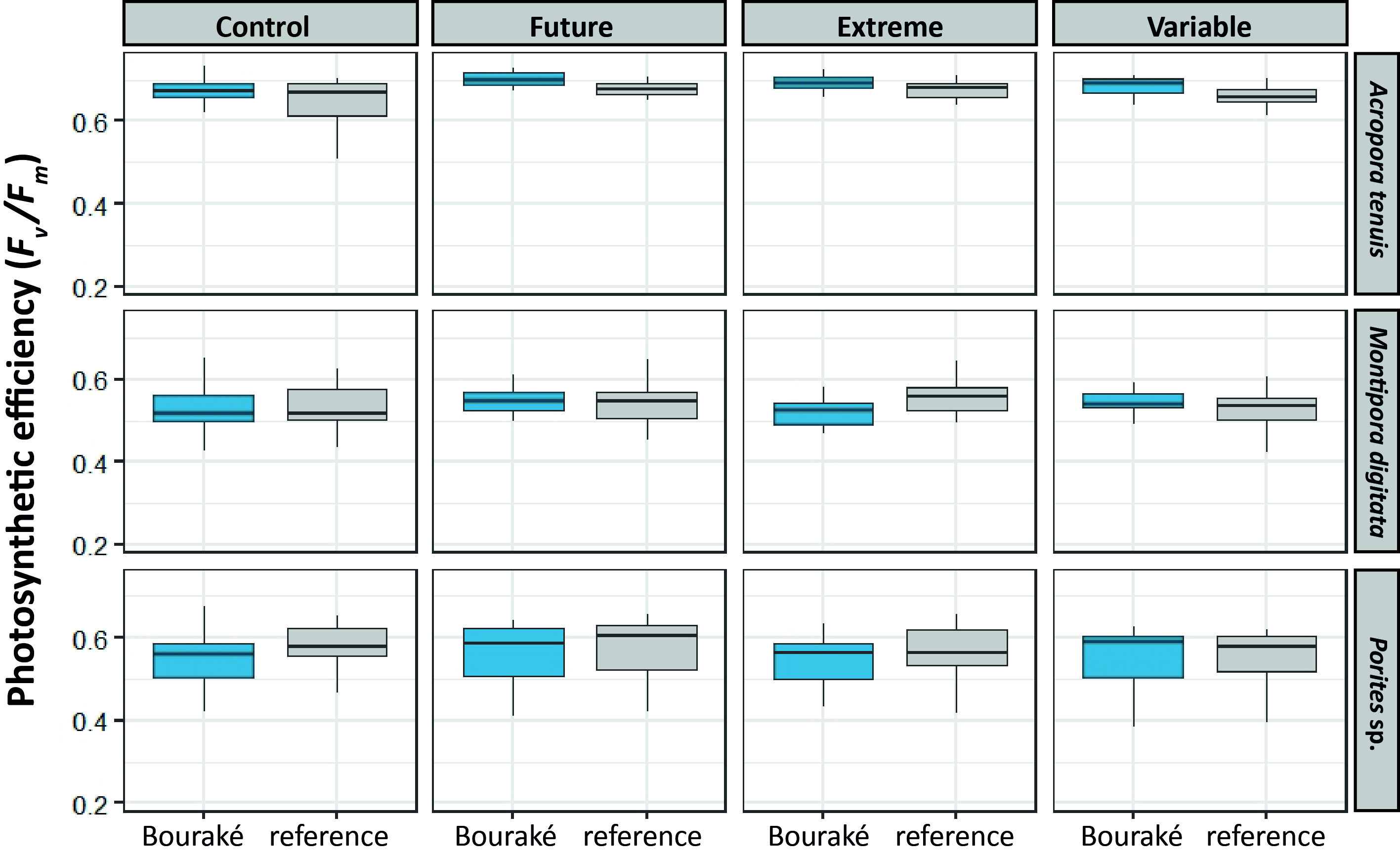
**

**Figure A4.** Photosynthetic efficiency (*F_v_/F_m_*) of corals from Bouraké (in blue) and reference (in grey) site measured after 100 days of incubation at four pH conditions (Control, pH_NBS_ 8.11; Future, pH_NBS_ 7.76; Extreme, pH_NBS_ 7.54; and Variable, pH_NBS_ 7.56-8.07). Data are median ± 25th and 75th percentiles (n=12-16, depending on species and pH condition ; see Table A7 for all post hoc comparisons).


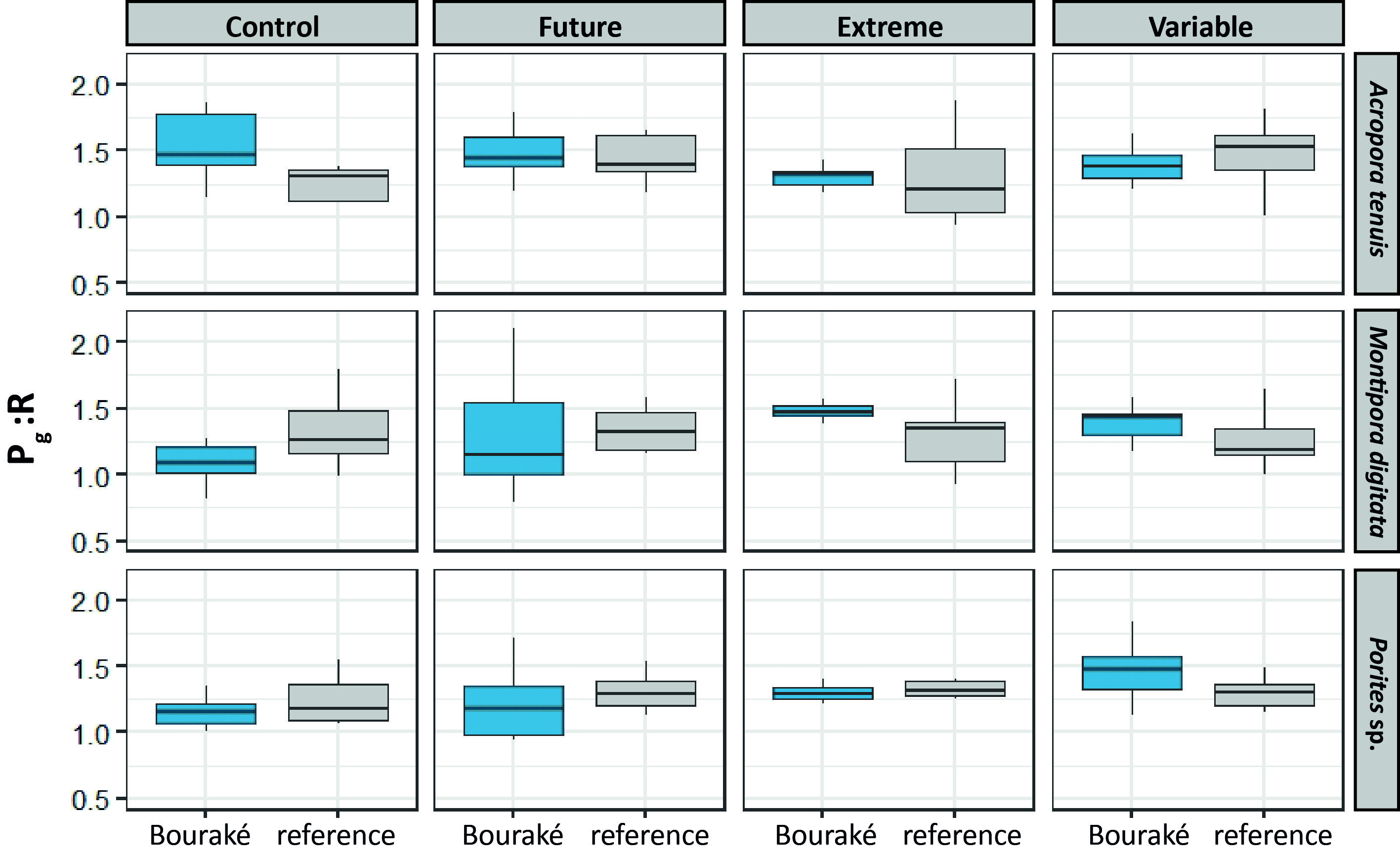


**Figure A5.** Gross photosynthesis to respiration ratio (P_g_:R) of corals from Bouraké (in blue) and the reference (in grey) site incubated at four pH conditions (Control, pH_NBS_ 8.11; Future, pH_NBS_ 7.76; Extreme, pH_NBS_ 7.54; and Variable, pH_NBS_ 7.56-8.07). Data are median ± 25th and 75th percentiles (n = 7 ; see Table A7 for all post hoc comparisons).


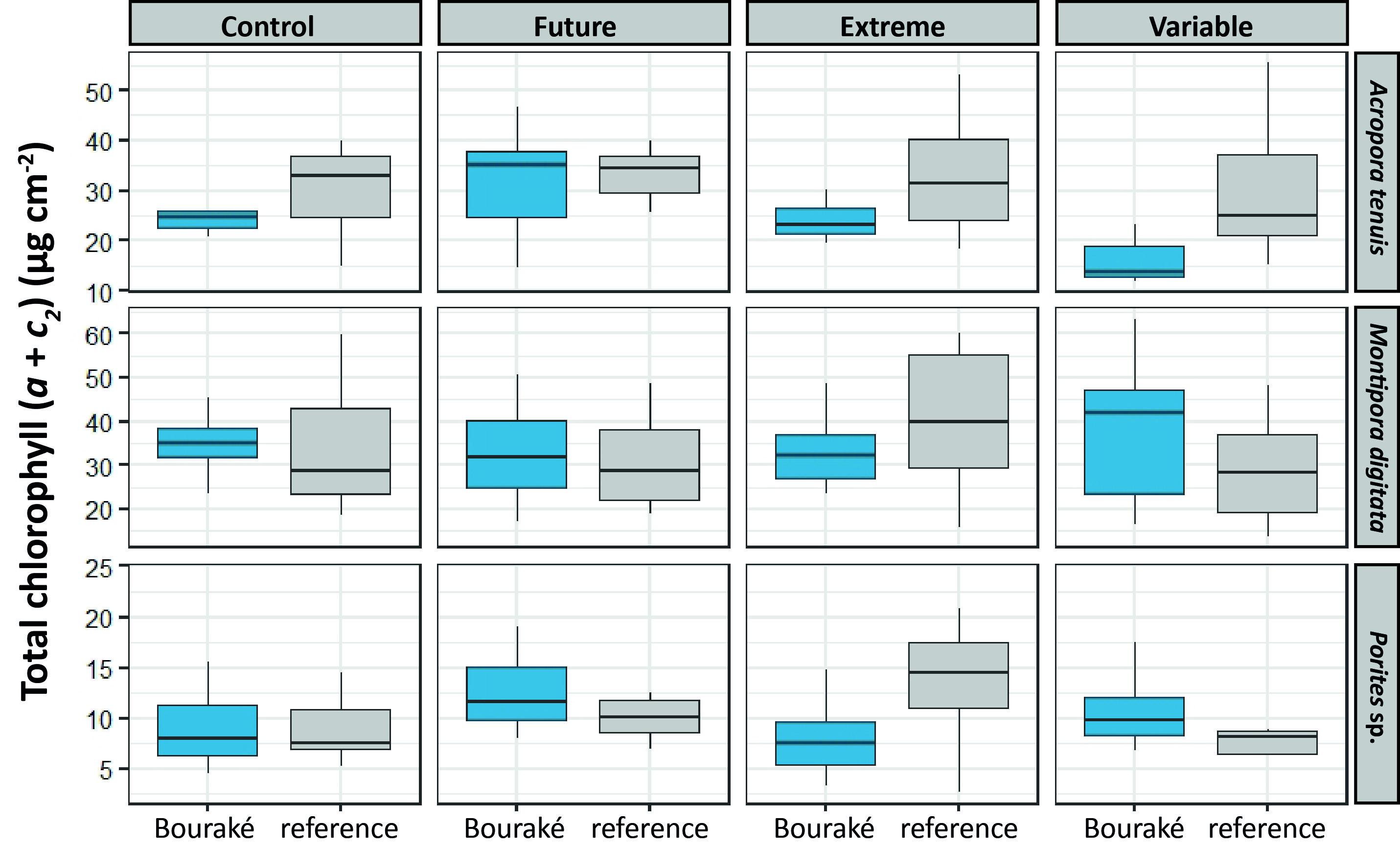


**Figure A6.** Total chlorophyll (*a* + *c*_2_) content of corals from Bouraké (in blue) and reference (in grey) site after 100 days of incubation at four pH conditions (Control, pH_NBS_ 8.11; Future, pH_NBS_ 7.76; Extreme, pH_NBS_ 7.54; and Variable, pH_NBS_ 7.56-8.07). Data are median ± 25th and 75th percentiles (n = 7; see Table A7 for all post hoc comparisons)

**
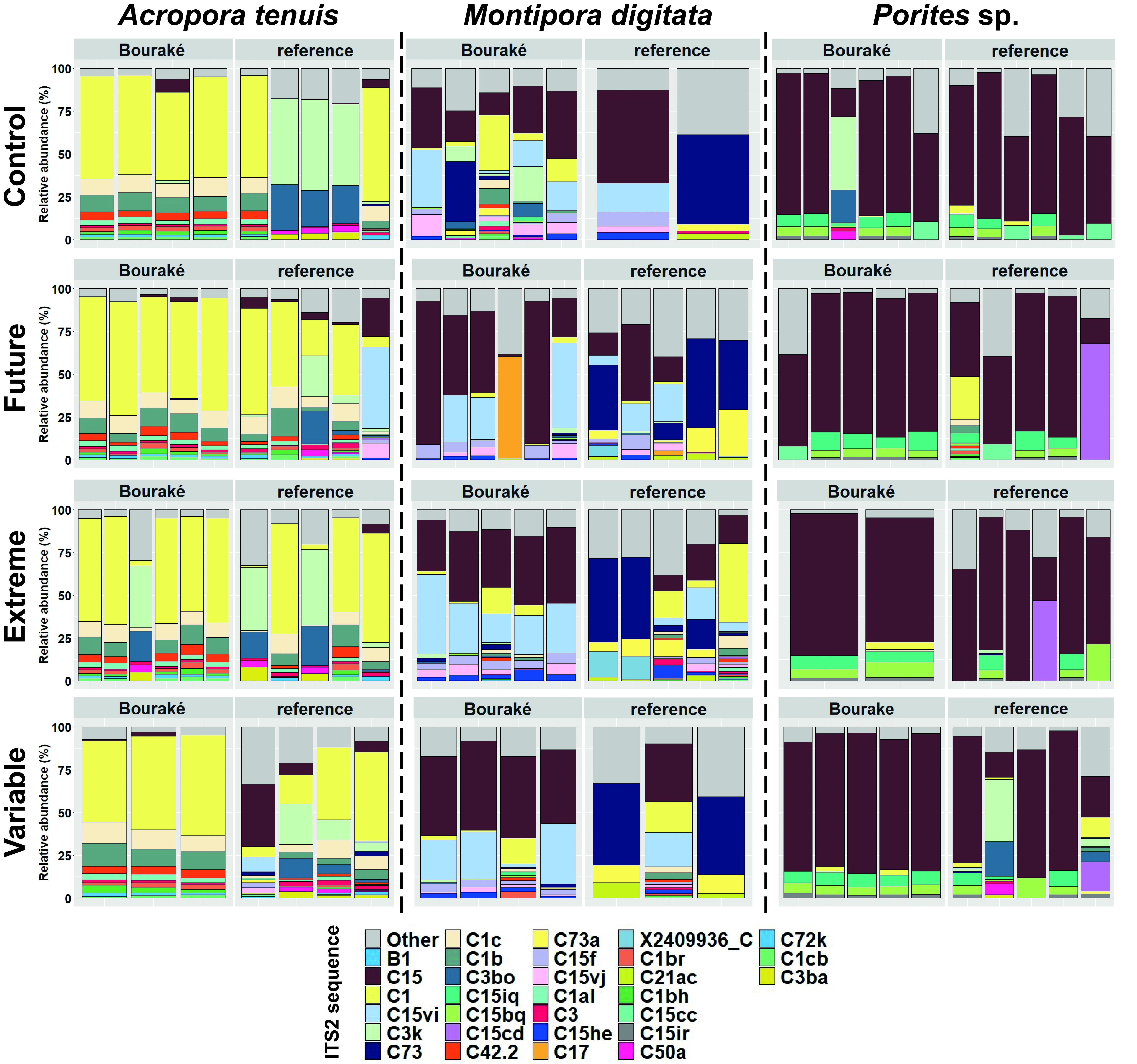
**

**Figure A7.** Relative abundance (%) ITS2 sequence of corals from Bouraké and the reference site after 100 days of incubation at four pH conditions (Control, pH_NBS_ 8.11; Future, pH_NBS_ 7.76; Extreme, pH_NBS_ 7.54; and Variable, pH_NBS_ 7.56-8.07). Within each cell of this 3-by-4 matrix, samples are plotted as stacked bar charts with a single column representing a sample from a specific origin: Bouraké (left) and reference (right). Only the 30 most abundant ITS2 sequences have been assigned colors. Other low abundance recovered sequences are in grey. Sequences with designated names (e.g., B1, C15, or C1) refer to sequences previously characterized in the literature or that have been run through the SymPortal analytical framework. Less common sequences and those that have not been used to characterize ITS2-type profiles are designated using a unique database ID and their associated clade/genera (e.g., X2409936_C ; the latter letter referring to *Cladocopium* genera (Clade C)).

**
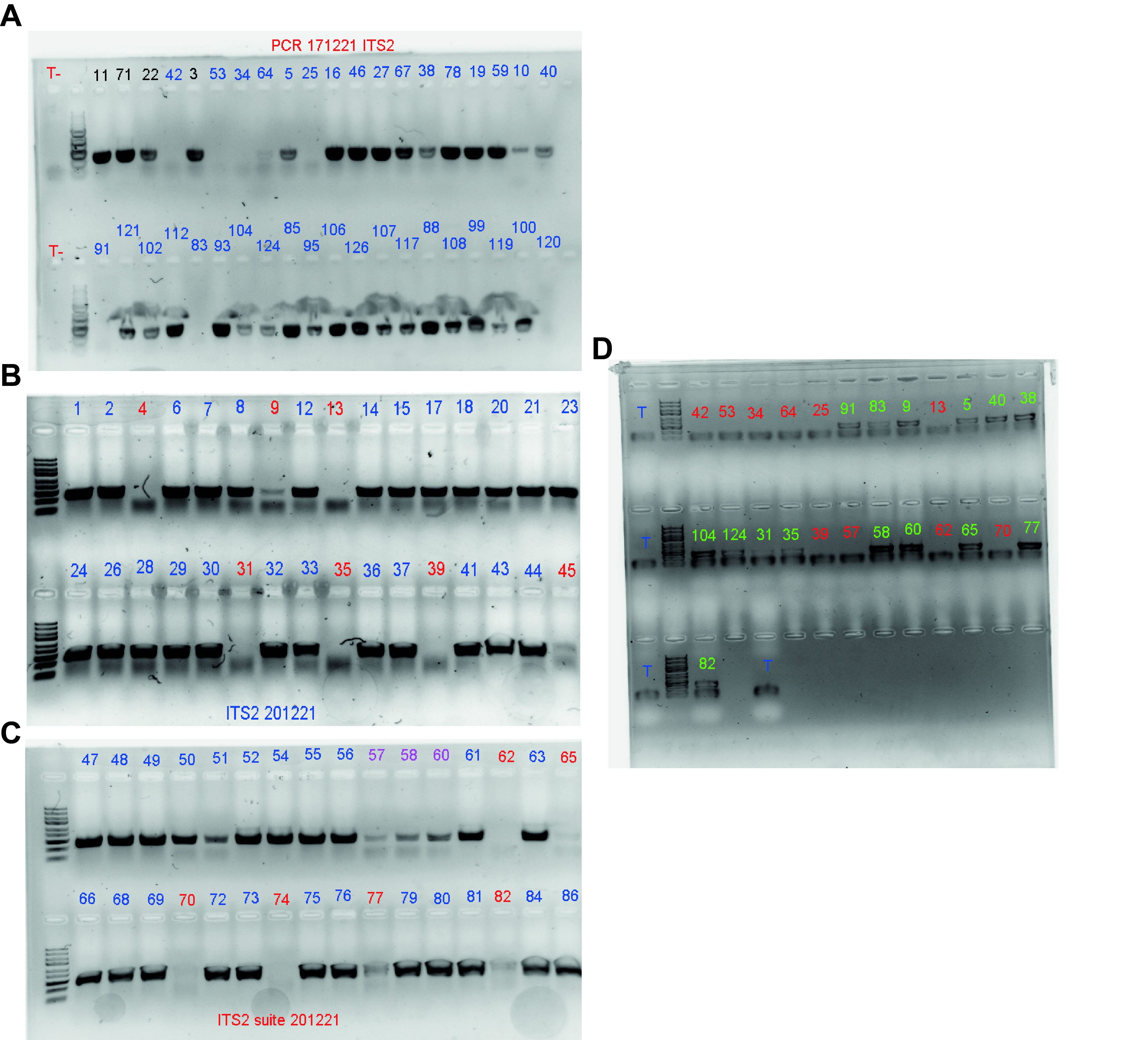
**

**Figure A8.** Control gels of the PCR. Negative controls are represented with a T. First, we ran random PCR samples on gels (A); after noticing some unsuccessful PCR, we checked all PCR samples from the same session on gels (B & C). The unsuccessful PCR were run again during a new session with adjustments of the hybridation time, and were checked again on gels (D). The remaining unsuccessful PCR (n=10), and those that did not pass the AGRF quality control were removed from the study (n=4) leading to a total of 112 samples for ITS2 sequencing.

**Table A1.** Time table used by the IKS logger (Timer function) in the Variable condition to mimic the natural pH variation measured at Bouraké. For each time step, IKS activated the pump that supplied seawater from the sump to the experimental tanks at the desired value.

| **Step** | **Time start** | **Time end** | **pH sump** |
| --- | --- | --- | --- |
| 1 | 00:00:00 | 00:30:00 | 7.6 |
| 2 | 00:30:00 | 01:00:00 | 7.7 |
| 3 | 01:00:00 | 01:30:00 | 7.8 |
| 4 | 01:30:00 | 02:30:00 | 7.9 |
| 5 | 02:00:00 | 05:00:00 | 8.1 |
| 6 | 04:30:00 | 05:30:00 | 7.9 |
| 7 | 05:30:00 | 06:30:00 | 7.8 |
| 8 | 06:30:00 | 07:30:00 | 7.7 |
| 9 | 07:30:00 | 08:30:00 | 7.6 |
| 10 | 08:30:00 | 12:00:00 | 7.4 |
| 11 | 12:00:00 | 12:30:00 | 7.6 |
| 12 | 12:30:00 | 13:00:00 | 7.7 |
| 13 | 13:00:00 | 13:30:00 | 7.8 |
| 14 | 13:30:00 | 14:30:00 | 7.9 |
| 15 | 14:00:00 | 17:00:00 | 8.1 |
| 16 | 16:30:00 | 17:30:00 | 7.9 |
| 17 | 17:30:00 | 18:30:00 | 7.8 |
| 18 | 18:30:00 | 19:00:00 | 7.7 |
| 19 | 19:00:00 | 21:30:00 | 7.6 |
| 20 | 21:00:00 | 00:00:00 | 7.4 |
|  |  |  |  |

**Table A2.** Percentage of irradiance intensity for each of the LED light spectra during a diel cycle (from 6 am to 5 pm).

| \| **Hour** \| **Blue** \| **Royal blue** \| **Cool white** \| **Red** \| **True green** \| **Hyper red** \| **Yellow** \| **Neutral white** \| \| --- \| --- \| --- \| --- \| --- \| --- \| --- \| --- \| --- \| \| \| 05:00:00 \| 0 \| 0 \| 0 \| 0 \| 0 \| 0 \| 0 \| 0 \| \| 06:00:00 \| 10 \| 10 \| 20 \| 20 \| 0 \| 0 \| 0 \| 20 \| \| 07:00:00 \| 20 \| 20 \| 40 \| 30 \| 0 \| 0 \| 0 \| 25 \| \| 08:00:00 \| 30 \| 30 \| 60 \| 45 \| 10 \| 0 \| 10 \| 30 \| \| 10:00:00 \| 40 \| 40 \| 80 \| 60 \| 10 \| 10 \| 15 \| 40 \| \| 11:00:00 \| 80 \| 80 \| 90 \| 60 \| 15 \| 25 \| 15 \| 60 \| \| 12:00:00 \| 100 \| 100 \| 100 \| 60 \| 20 \| 40 \| 20 \| 80 \| \| 14:30:00 \| 100 \| 100 \| 100 \| 60 \| 20 \| 40 \| 20 \| 80 \| \| 16:00:00 \| 80 \| 80 \| 40 \| 20 \| 10 \| 20 \| 10 \| 40 \| \| 16:30:00 \| 60 \| 40 \| 20 \| 10 \| 0 \| 0 \| 0 \| 0 \| \| 17:00:00 \| 0 \| 0 \| 0 \| 0 \| 0 \| 0 \| 0 \| 0 \| |  |  |  |  |  |  |  |  |
| --- | --- | --- | --- | --- | --- | --- | --- | --- | --- | --- | --- | --- | --- | --- | --- | --- | --- | --- | --- | --- | --- | --- | --- | --- | --- | --- | --- | --- | --- | --- | --- | --- | --- | --- | --- | --- | --- | --- | --- | --- | --- | --- | --- | --- | --- | --- | --- | --- | --- | --- | --- | --- | --- | --- | --- | --- | --- | --- | --- | --- | --- | --- | --- | --- | --- | --- | --- | --- | --- | --- | --- | --- | --- | --- | --- | --- | --- | --- | --- | --- | --- | --- | --- | --- | --- | --- | --- | --- | --- | --- | --- | --- | --- | --- | --- | --- | --- | --- | --- | --- | --- | --- | --- | --- | --- | --- | --- | --- | --- | --- | --- | --- | --- | --- | --- | --- |

**Table A3.** Seawater carbonate chemistry daily measurements during the 100 days of incubation at four pH conditions (Control, pH_NBS_ 8.11; Future, pH_NBS_ 7.76; Extreme, pH_NBS_ 7.54; and Variable, pH_NBS_ 7.56-8.07), and for each replicate tank (A, B and C). Seawater carbonate chemistry was calculated using mean values for *A*_T_ (2187, 2204, 2202, and 2202 µmol kg^-1^ for Control, Future, Extreme, and Variable, respectively) and the mean salinity value of 35.61.

**Measured**  **Calculated**

**Temp pH *p*CO_2_ DIC HCO_3_^-^ CO_3_^2-^ Ω_arag_**

(°C) (NBS) (µatm) (µmol kg^-1^) (µmol kg^-1^) (µmol kg^-1^)

**Control**

Tank A Mean 26.29 8.11 484 1932 1737 182 2.89

SD 0.48 0.05 72 29 45 18 0.29

Min 24.20 7.96 348 1870 1640 136 2.17

Max 27.30 8.22 719 2007 1851 220 3.49

Tank B Mean 26.15 8.11 472 1916 1721 182 2.89

SD 0.49 0.05 67 30 46 18 0.29

Min 24.20 8.00 299 1825 1579 148 2.34

Max 27.30 8.27 629 1972 1806 238 3.78

Tank C Mean 26.26 8.11 481 1930 1734 183 2.90

SD 0.49 0.06 77 32 50 20 0.31

Min 24.20 7.97 326 1850 1609 141 2.24

Max 27.50 8.24 693 1999 1839 232 3.69

**Future**

Tank A Mean 26.15 7.75 1252 2096 1971 90 1.43

SD 0.54 0.06 172 21 27 11 0.17

Min 24.20 7.63 797 2027 1878 69 1.09

Max 27.50 7.92 1673 2139 2024 127 2.02

Tank B Mean 26.15 7.78 1184 2104 1974 97 1.54

SD 0.53 0.08 220 21 40 16 0.25

Min 24.20 7.65 625 2003 1832 73 1.16

Max 27.20 8.01 1609 2151 2033 154 2.44

Tank C Mean 26.24 7.77 1186 2091 1964 95 1.51

SD 0.49 0.07 192 25 33 13 0.21

Min 24.20 7.66 732 2018 1862 75 1.19

Max 27.20 7.95 1540 2132 2015 136 2.16

**Extreme**

Tank A Mean 26.31 7.51 2262 2183 2066 55 0.88

SD 0.48 0.06 354 21 19 8 0.12

Min 24.30 7.31 1377 2120 2001 35 0.55

Max 28.00 7.71 3626 2252 2117 81 1.29

Tank B Mean 26.30 7.55 2070 2169 2051 61 0.97

SD 0.53 0.09 430 32 35 14 0.22

Min 23.90 7.38 686 2011 1851 41 0.66

Max 27.80 7.98 3051 2225 2100 141 2.24

Tank C Mean 26.38 7.54 2115 2168 2052 59 0.93

SD 0.49 0.07 335 22 22 9 0.14

**Table A3 continued**

Measured Calculated

**Temp pH *p*CO_2_ DIC HCO_3_^-^ CO_3_^2-^ Ω_arag_**

(°C) (NBS) (µatm) (µmol kg^-1^) (µmol kg^-1^) (µmol kg^-1^)

Min 24.30 7.38 1168 2091 1966 41 0.64

Max 28.30 7.77 3113 2222 2097 93 1.47

**Variable**

Tank A Mean 26.32 7.74 1456 2093 1954 99 1.57

SD 0.60 0.23 730 94 124 50 0.79

Min 24.30 7.39 371 1893 1671 42 0.66

Max 28.30 8.20 2979 2222 2098 212 3.36

Tank B Mean 26.39 7.76 1379 2088 1949 102 1.62

SD 0.60 0.22 670 91 121 48 0.77

Min 24.20 7.40 404 1910 1696 42 0.67

Max 28.00 8.17 2948 2222 2098 203 3.22

Tank C Mean 26.46 7.75 1446 2089 1948 101 1.61

SD 0.52 0.24 733 96 128 51 0.82

Min 24.40 7.37 412 1912 1701 40 0.63

Max 28.10 8.16 3179 2229 2102 200 3.17

**Table A4.** Seawater daily measurements during 100 days in the 6 tank sumps (i.e., pH_T_ 7.4, 7.6, 7.7, 7.8, 7.9 and 8.1) that alimented the experimental tanks.

**Sump tank**  **Temp (°C) pH_NBS_**

**7.4** Mean 26.26 7.43

SD 0.47 0.07

**7.6** Mean 26.50 7.57

SD 0.90 0.06

**7.7** Mean 26.10 7.68

SD 0.60 0.08

**7.8** Mean 26.51 7.82

SD 0.88 0.03

**7.9** Mean 26.40 7.90

SD 0.87 0.03

**8.1** Mean 26.05 8.06

SD 0.53 0.04

**Table A5.** Aligned Rank Transformed (ART) ANOVAs (Type III) summary with Kenward-Roger method of linear mixed effects model (LMER) on the effects of temperature, pH and nutrients between pH conditions (Control, pH_NBS_ 8.11; Future, pH_NBS_ 7.76; Extreme, pH_NBS_ 7.54; and Variable, pH_NBS_ 7.56-8.07). Significant values are in bold (*p* < 0.05)

**Fixed factors** *df F p* **Random factors** Variance SD

Temperature (3, 1206) 9.855 **<0.001** Tank 0.001 0.039

pH (3, 1206) 889.130 **<0.001** Tank <0.001 0.010

NO_x_ (3, 90) 0.588 0.624 Tank 0.000 0.000

PO_4_^3-^ (3, 90) 0.038 0.990 Tank 0.000 0.000

Si(OH)_4_ (3, 90) 0.040 0.989 Tank 0.000 0.000

**Table A6**. Seawater nutrient contents measured two times a month during the 100 days of incubation at four pH conditions (Control, pH_NBS_ 8.11; Future, pH_NBS_ 7.76; Extreme, pH_NBS_ 7.54; and Variable, pH_NBS_ 7.56-8.07) , and for each replicated tank (A, B and C).

**pH condition NO_x_ PO_4_^3-^ Si(OH)_4_**

(µmol L^-1^) (µmol L^-1^) (µmol L^-1^)

**Control** Tank A Mean 0.56 0.30 2.86

SD 0.20 0.04 1.07

Min 0.31 0.26 1.86

Max 0.84 0.35 4.92

Tank B Mean 0.52 0.30 2.71

SD 0.17 0.06 1.20

Min 0.28 0.19 1.49

Max 0.79 0.36 5.12

Tank C Mean 0.68 0.30 2.92

SD 0.26 0.04 1.39

Min 0.35 0.25 1.18

Max 1.08 0.37 5.65

**Future** Tank A Mean 0.71 0.31 3.00

SD 0.31 0.07 1.44

Min 0.33 0.22 1.07

Max 1.21 0.41 5.63

Tank B Mean 0.64 0.30 2.83

SD 0.19 0.05 1.01

Min 0.36 0.22 1.68

Max 0.87 0.38 4.54

Tank C Mean 0.65 0.31 2.84

SD 0.26 0.05 1.22

Min 0.40 0.21 1.01

Max 1.14 0.39 4.70

**Extreme** Tank A Mean 0.63 0.30 2.85

SD 0.21 0.03 1.27

Min 0.39 0.25 1.01

Max 0.93 0.33 5.31

Tank B Mean 0.68 0.30 2.97

SD 0.23 0.05 1.37

Min 0.37 0.24 0.85

Max 1.06 0.41 5.58

Tank C Mean 0.57 0.31 2.56

SD 0.18 0.04 1.11

Min 0.21 0.25 1.03

Max 0.76 0.38 4.66

**Variable** Tank A Mean 0.60 0.30 2.79

SD 0.15 0.03 0.89

Min 0.40 0.24 1.81

Max 0.90 0.34 4.27

Tank B Mean 0.67 0.30 2.80

SD 0.17 0.04 1.25

Min 0.48 0.26 1.63

Max 0.93 0.39 5.13

Tank C Mean 0.68 0.31 2.78

SD 0.29 0.04 1.17

Min 0.32 0.27 1.76

Max 1.23 0.39 5.12

**Table A7.** Two-way ANOVAs (type III) summary with Satterthwaite’s method of linear mixed effect model (LMER) on the effects of colony origin (Bouraké and reference), pH conditions (Control, pH_NBS_ 8.11; Future, pH_NBS_ 7.76; Extreme, pH_NBS_ 7.54; and Variable, pH_NBS_ 7.56-8.07), and their interactions for each coral species and for each physiological measurement. When a significant effect was found on pH (4 levels: Control, Future, Extreme and Variable), we used a post-hoc Tukey HSD. Non-parametric two-way Aligned Rank Transformed (ART) ANOVA (Type III) with Kenward-Roger method followed by a Bonferroni p-levels adjusted post hoc was used for *F_v_/F_m_*. For simplicity, Control = 8.1; Future = 7.8; Extreme = 7.5; Variable = Var; R = reference; B = Bouraké. Significant values are in bold (*p* < 0.05).

*Species Fixed factors df F p Post hoc*

**A) Growth rate**

*A. tenuis* Origin 1 9.334 **0.003**

pH 3 3.429 **0.020** 8.1 > Var (*p* < 0.05)

Origin x pH 3 0.809 0.491

Residuals 111

*Random factor Variance SD*

Tank 0.000 0.000

*Fixed factors df F p Post hoc*

*M. digitata* Origin 1 27.563 <**0.001**

pH 3 2.203 0.092

Origin x pH 3 0.760 0.519

Residuals 107

*Random factor Variance SD*

Tank 0.000 0.000

*Fixed factors df F p Post hoc*

*Porites* sp Origin 1 18.407 <**0.001**

pH 3 2.787 **0.044** 8.1 > 7.5 (*p* < 0.05)

Origin x pH 3 0.843 0.473

Residuals 110

*Random factor Variance SD*

Tank 0.000 0.000

**B) *F_v_/F_m_*** *Fixed factors df F p Post hoc*

*A. tenuis* Origin 1 11.213 **0.001**

pH 3 3.517 **0.018** 7.8 = 7.5 > 8.1 (*p* < 0.05)

Origin X pH 3 0.330 0.803

Residuals 108

*Random factor Variance SD*

Tank <0.001 0.007

**Table A7 continued**

*Species Fixed factor df F p Post hoc*

*M. digitata* Origin 1 0.132 0.717

pH 3 0.615 0.606

Origin X pH 3 1.660 0.180

Residuals 107

*Random factor Variance SD*

Tank 0.000 0.000

*Fixed factors df F p Post hoc*

*Porites* sp Origin 1 1.207 0.274

pH 3 0.691 0.559

Origin X pH 3 0.222 0.881

Residuals 107

*Random factor Variance SD*

Tank <0.001 0.012

**C) ETR_max_** *Fixed factors df F p Post hoc*

*A. tenuis* Origin 1 16.449 <**0.001**

pH 3 1.038 0.379

Origin x pH 3 0.134 0.939

Residuals 108

*Random factor Variance SD*

Tank 0.000 0.000

*Fixed factors df F p Post hoc*

*M. digitata* Origin 1 0.032 0.858

pH 3 0.890 0.445

Origin x pH 3 0.874 0.457

Residuals 107

*Random factor Variance SD*

Tank 1.422 1.192

*Fixed factors df F p Post hoc*

*Porites* sp. Origin 1 0.051 0.822

pH 3 0.647 0.586

Origin x pH 3 0.602 0.615

Residuals 107

*Random factor Variance SD*

Tank 0.000 0.000

**D) P_g_** *Fixed factors df F p Post hoc*

*A. tenuis* Origin 1 3.609 0.063

pH 3 4.375 **0.008** 8.1 < 7.8 (*p* < 0.01)

Origin x pH 3 1.265 0.297

Residuals 48

*Random factor Variance SD*

Tank 0.000 0.000

**Table A7 continued**

*Species Fixed factor df F p Post hoc*

*M. digitata* Origin 1 0.376 0.543

pH 3 2.001 0.126

Origin x pH 3 2.240 0.096

Residuals 48

*Random factor Variance SD*

Tank 0.000 0.000

*Fixed factors df F p Post hoc*

*Porites* sp. Origin 1 7.277 **0.010**

pH 3 8.503 **<0.001** 8.1 = 7.8 < Var **=** 7.5 (*p* < 0.04)

Origin x pH 3 2.771 0.052

Residuals 48

*Random factor Variance SD*

Tank 0.000 0.000

**E) R_dark_** *Fixed factors df F p Post hoc*

*A. tenuis* Origin 1 5.585 **0.022**

pH 3 5.538 **0.002** Var = 8.1 < 7.8 = 7.5 (*p* < 0.03)

Origin x pH 3 1.211 0.316

Residuals 48

*Random factor Variance SD*

Tank 0.000 0.000

*Fixed factors df F p Post hoc*

*M. digitata* Origin 1 0.133 0.717

pH 3 1.786 0.163

Origin x pH 3 0.862 0.468

Residuals 48

*Random factor Variance SD*

Tank <0.001 0.013

*Fixed factors df F p Post hoc*

*Porites* sp. Origin 1 7.436 **0.009**

pH 3 3.676 **0.018** 8.1 < 7.5 (p < 0.02)

Origin x pH 3 1.067 0.372

Residuals 48

*Random factor Variance SD*

Tank 0.000 0.000

**F) P_g_ : R** *Fixed factors df F p Post hoc*

*A. tenuis* Origin 1 0.874 0.355

pH 3 1.658 0.188

Origin x pH 3 2.274 0.092

Residuals 48

*Random factor Variance SD*

Tank 0.000 0.000

**Table A7 continued**

*Species Fixed factor df F p Post hoc*

*M. digitata* Origin 1 0.143 0.706

pH 3 0.579 0.632

Origin x pH 3 1.285 0.291

Residuals 48

*Random factor Variance SD*

Tank <0.001 0.023

*Fixed factors df F p Post hoc*

*Porites* sp Origin 1 0.012 0.912

pH 3 2.728 0.054

Origin x pH 3 1.132 0.346

Residuals 48

*Random factor Variance SD*

Tank 0.000 0.000

**G) Symbiont** *Fixed factors df F p Post hoc*

*A. tenuis* Origin 1 5.870 **0.015**

pH 3 2.700 **0.044** 7.8 > Var (*p* < 0.05)

Origin x pH 3 2.860 **0.035** R 7.5 > B Var (*p* < 0.05)

Residuals 54

*Random factor Variance SD*

Tank 0.000 0.000

*Fixed factors df F p Post hoc*

*M. digitata* Origin 1 0.005 0.946

pH 3 1.987 0.113

Origin x pH 3 1.335 0.261

Residuals 48

*Random factor Variance SD*

Tank 0.000 0.000

*Fixed factors df F p Post hoc*

*Porites* sp Origin 1 2.707 0.100

pH 3 1.237 0.294

Origin x pH 3 2.622 **0.049** R 7.5 > B 7.5 (*p* < 0.05)

Residuals 54

*Random factor Variance SD*

Tank >9.999 >9.999

**H) Total chl** *Fixed factors df F p Post hoc*

*A. tenuis* Origin 1 10.695 **0.002**

pH 3 2.652 0.058

Origin x pH 3 1.319 0.278

Residuals 54

*Random factor Variance SD*

Tank 0.000 0.000

**Table A7 continued**

*Species Fixed factor df F p Post hoc*

*M. digitata* Origin 1 0.064 0.802

pH 3 0.378 0.770

Origin:pH 3 0.872 0.462

Residuals 48

*Random factor Variance SD*

Tank 0.000 0.000

*Fixed factors df F p Post hoc*

*Porites* sp Origin 1 0.316 0.576

pH 3 1.560 0.210

Origin:pH 3 3.155 **0.032**

Residuals 54

*Random factor Variance SD*

Tank 0.000 0.000

**I) Proteins** *Fixed factors df F p Post hoc*

*A. tenuis* Origin 1 1.047 0.311

pH 3 3.263 **0.029** 8.1 > Var (*p* < 0.04)

Origin:pH 3 0.522 0.669

Residuals 48

*Random factor Variance SD*

Tank 0.000 0.000

*Fixed factors df F p Post hoc*

*M. digitata* Origin 1 0.945 0.336

pH 3 1.928 0.138

Origin:pH 3 0.439 0.726

Residuals 48

*Random factor Variance SD*

Tank 0.049 0.221

*Fixed factors df F p Post hoc*

Porites sp Origin 1 0.654 0.423

pH 3 5.782 **0.002** 8.1 > Var = 7.7 (*p* < 0.01)

Origin:pH 2 0.848 0.436

Residuals 40

*Random factor Variance SD*

Tank 0.000 0.000
